# Amphiphilic Oligonucleotide Derivatives as a Tool to Study DNA Repair Proteins

**DOI:** 10.3390/ijms26157078

**Published:** 2025-07-23

**Authors:** Svetlana N. Khodyreva, Alexandra A. Yamskikh, Ekaterina S. Ilina, Mikhail M. Kutuzov, Ekaterina A. Belousova, Maxim S. Kupryushkin, Timofey D. Zharkov, Olga A. Koval, Sofia P. Zvereva, Olga I. Lavrik

**Affiliations:** 1 Institute of Chemical Biology and Fundamental Medicine, Siberian Branch of the Russian Academy of Sciences, 8 Akad. Lavrentyeva Ave., 630090 Novosibirsk, Russia; a.yamskikh@g.nsu.ru (A.A.Y.); katya.plekhanova@gmail.com (E.S.I.); kutuzov.mm@mail.ru (M.M.K.); rina@niboch.nsc.ru (E.A.B.); kuprummax@niboch.nsc.ru (M.S.K.); timazharkov74@gmail.com (T.D.Z.); olgaakoval@yandex.ru (O.A.K.); zvereksonik@gmail.com (S.P.Z.); 2 Department of Natural Sciences, Novosibirsk State University, 2 Pirogova Str., 630090 Novosibirsk, Russia; 3 Sechenov Institute of Evolutionary Physiology and Biochemistry, Russian Academy of Sciences, 44 Thorez pr., 194223 St. Petersburg, Russia

**Keywords:** lipophilic substituents in DNA, PARP1, PARP2, PARP3, poly(ADP-ribose), 5′dRP lyase, SH-SY5Y cells

## Abstract

Modified oligonucleotides (oligos) are widely used as convenient tools in many scientific fields, including biomedical applications and therapies. In particular, oligos with lipophilic groups attached to the backbone ensure penetration of the cell membrane without the need for transfection. This study examines the interaction between amphiphilic DNA duplexes, in which one of the chains contains a lipophilic substituent, and several DNA repair proteins, particularly DNA-damage-dependent PARPs, using various biochemical approaches. DNA with a lipophilic substituent (LS-DNA) demonstrates more efficient binding with DNA damage activated poly(AD-ribose) polymerases 1-3 (PARP1, PARP2, PARP3) and DNA polymerase β. Chemically reactive LS-DNA derivatives containing a photoactivatable nucleotide (photo-LS-DNAs) or a 5′ deoxyribose phosphate (dRP) group in the vicinity of double-strand breaks (DSBs) are used for the affinity labelling of PARPs and other proteins in several whole-cell extracts of human cells. In particular, photo-LS-DNAs are used to track the level of Ku antigen in the extracts of neuron-like differentiated SH-SY5Y, undifferentiated SH-SY5Y, and olfactory epithelial cells. In vitro, PARP1–PARP3 are shown to be able to slowly excise the 5′ dRP group at DSBs. LS-DNAs can activate PARP1 and PARP2 for autoPARylation, albeit less effectively than regular DNA duplexes.

## 1. Introduction

Modified oligonucleotides are widely used as convenient tools in many scientific fields, including therapeutic and biomedical applications [1,2,3]. The attachment of lipophilic groups to the oligonucleotide backbone, e.g., alkyl-containing groups, cholesterol, fatty acids, and lipids, is a promising approach to ensure penetration of the functionalised macromolecule through the cell membrane [4,5,6,7,8,9]. A cholesterol-coupled oligonucleotide was previously used as part of the so-called AsiDNA™, which was developed to sensitise tumour cells to genotoxic therapy by acting as a decoy to disorganise the DNA damage response [4,5,10]. In this work, a DNA duplex containing the cholesterol-conjugated oligonucleotide was used as a control DNA duplex in a study of the effect of substituent type on the interaction of DNAs with proteins of interest. Previously, dodecyl-containing nucleic acid structures with phosphoramidate or non-nucleotide modifications have been shown to penetrate cells in the absence of transfectants [6,7,8]. A phosphorylguanidine (PG) substituent on the internucleotide phosphate is an effective modification to prevent the hydrolysis of nucleic acids by nucleases in organisms [11]. In addition, dynamic light scattering has shown that oligonucleotides (ONs) bearing triazinyl phosphoramidate with dodecyl residues are able to self-assemble into micelle-like particles [8]. This ability to self-assemble is an extremely important property of macromolecules that influences their behaviour in living systems.

Here, we studied the interaction of amphiphilic DNA duplexes, in which one of the chains contained a lipophilic substituent, with several DNA repair proteins, in particular with PARPs, which are activated by DNA damage.

Oligonucleotides with lipophilic substituents contained (a) the triazinylphosphoramidate group functionalised with two dodecyl substituents near the 3′ end; (b) the same modification supplemented with a phosphorylguanidine group near the 5′ end; or (c) a cholesterol residue attached to the 3′ end via a linker. In some experiments to determine how lipophilic substituents in DNA interfere with DNA–protein interactions, in the DNA duplexes studied, one chain contained a lipophilic group and the complementary chain was equipped with a chemically reactive group to covalently attach DNA to a protein and a ^32^P label to mark the cross-linked polypeptide chain. In all DNA duplexes used, the chemically reactive chain was the same, while the specificity of DNA–protein interaction was determined by the complementary chain carrying the substituents under investigation. The chemically reactive substituents were represented by a photoreactive group (FAP-dCMP residue at the 3′ position) or a 5′ deoxyribose phosphate group (5′dRP). Both groups have previously been used in different DNA contexts for the affinity modification (labelling) of DNA binding proteins [6,8,12,13]. Affinity labelling is an approach based on the analysis of covalent complexes formed between a protein and a chemically reactive analogue of a ligand, for example, the analysis of DNA–protein cross-links (DPCs). The photoreactive group was introduced into the DNA duplex by the template-dependent incorporation of exo-N-{2-[N-(4-azido-2,5-difluoro-3-chloropyridine-6-yl)-3-aminopropionyl]amnoethyl}-2ʹ-deoxycytidine-5′-monophosphate (FAP-dCMP) by DNA polymerase β (pol β) using the corresponding triphosphate as a substrate [13]. The photochemical properties of the photoreactive group introduced into DNA intermediates allowed for their excitation by near-UV light, which avoided stimulation of the intrinsic photoreactivity of nucleic acids and proteins.

**Figure 1:**
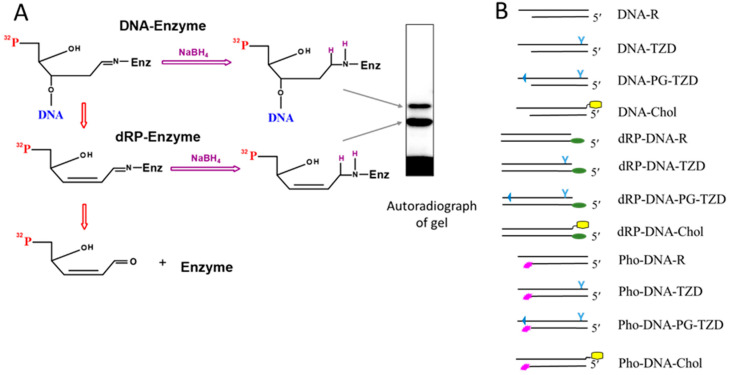
(**A**)—Scheme for the borohydride trapping of proteins on 5′dRP-DNA, followed by product analysis. (**B**)—Representation of the DNA with lipophilic substituents and their control DNA. The positions of the substituents are designated as follows: the phosphoryl guanidine group (PG)—triangles; the triazinylphosphoramidate groups functionalised with two dodecyl residues (TZDs)—symbol Y; the cholesterol substituent—yellow pentagons; the 5′dRP groups—green ovals; and the FAP-dCMP residues—magenta asterisks.

The 5′ ends with 5′dRP groups are incompatible with ligation, the final step in both single- and double-strand break repair. Mammalian DNA ligases 1, 3, and 4 lack the ability to remove this residue, so the 5′dRP end must be ‘trimmed’ by other proteins. The removal of 5′dRP occurs via the β-elimination mechanism, which is based on the formation of a transient aldimine intermediate, called a Schiff base, between the C1′ atom of deoxyribose and a primary amine group in the enzyme active site [14,15,16]. The deoxyribose in the 5′dRP group is in equilibrium between closed- and open-ring aldehyde forms, with the latter being capable of forming the Schiff base (Figure 1). The covalent enzyme–DNA intermediate is then converted to an enzyme–dRP intermediate after the elimination of the DNA chain. Both intermediates can be cross-linked, i.e., converted into an irreversible complex, by treatment with sodium borohydride (NaBH_4_) [14,15,16]. The 5′dRP residue can be used as a chemically reactive group for the capture of a protein in the covalent DNA–protein adduct. This approach has been used effectively to search for and identify proteins whose AP/5′dRP lyase activity was previously unknown [12,17].

A large number of mammalian proteins have AP/5′dRP lyase activity, in addition to its primary function [12,18,19,20,21,22,23,24,25,26,27,28].

It should be noted that 5′dRP groups can be toxic due to their ability to form cross-links with cellular proteins, including potentially undesirable targets such as proteins devoid of or exhibiting low 5′dRP lyase activity [16,18,19]. Nucleic-acid-interacting proteins rely mainly on electrostatic contacts to bind to nucleic acid and, therefore, contain positively charged amino acids in the corresponding regions, particularly lysines, whose primary amino groups can react with the open-ring form of deoxyribose in the vicinity of the 5′dRP residue.

Poly(ADP-ribose) (PAR) is a nucleic-acid-like polymer synthesised by poly(ADP-ribose) polymerases (PARPs). PARPs add ADP-ribose residues to target proteins as a post-translational modification synthesising PAR. PARP1, PARP2, and PARP3, members of a superfamily of 17 proteins, become catalytically active in response to DNA damage and add ADP-ribose residues to themselves (autoPARylation) or to other targets (heteroPARylation) [29]. In contrast to PARP1 and PARP2, PARP3 is a mono(ADP-ribose) transferase and can only attach one ADP-ribose residue. PARP1, PARP2, PARP3, and PAR are known to play roles in the regulation of several DNA repair processes [30,31].

The Ku antigen and PARP1 have been shown to efficiently bind to double-strand breaks in DNA with a high affinity and are key proteins involved in the earlier stages of canonical and alternative nonhomologous end joining (NHEJ) (C-NHEJ and Alt-NHEJ, respectively) [32,33,34,35,36,37]. The involvement of PARP2 and PARP3 in DSB repair has also been demonstrated [34,35,38,39,40].

We have previously shown that PARP1 and PARP2 exhibit 5′dRP lyase activity on the intermediates of base excision repair (BER), where the 5′dRP group is located at the single-strand nicks [21,41]. The Ku antigen has been reported to have strong 5′dRP lyase activity that excises the 5′dRP group near DS breaks, but not in BER substrates [26,27,28]. Pol β is known to be the major 5′dRP lyase in the BER pathway of DNA repair, removing 5′dRP at the nick, the structure formed by the APE1-catalysed hydrolysis of AP sites [16,42,43,44]. Pol β has been reported to have weak 5′dRP lyase activity at DS breaks [26,27,28]. A more comprehensive study of pol β 5′dR lyase activity reports that the enzyme is 60-fold more active on BER substrates than within a short 5′ overhang of DSB substrates [45]. However, this is a rather significant activity, approximately 80-fold higher than the activity of pol theta (pol θ) involved in the so-called pol θ-mediated end joining (TMEJ), the specific pathway of Alt-NHEJ [37,45,46,47,48].

We have recently shown that, in vitro, PARP1 and the Ku antigen are able to bind 30-mer DNA duplexes, where one chain is represented by an oligonucleotide bearing the triazinylphosphoramidate group functionalised with two dodecyl substituents (TZDs) [8], but details of the influence of lipophilic groups on the functionality of modified DNA in the case of other DNA repair proteins require further investigation. In light of these findings, we were curious to explore how lipophilic substituents at the internucleotide phosphates of oligonucleotides influence the interaction of these DNA duplexes with some DNA binding proteins involved in DNA repair, specifically in NHEJ.

Repurposing PARP inhibitors for non-oncological use could facilitate the development of novel therapeutic strategies, particularly in view of the apparent involvement of PARP1 in neurological disorders (NDs). PARP1 hyperactivity has been associated with neuronal death and ND progression. Using PARP inhibitors in neurology requires appropriate cell models, and SH-SY5Y cells are an attractive option, as they can be differentiated into cells that morphologically resemble primary neurons and express increased levels of neuron-specific markers [49,50]. This differentiation is maintained by the specific modulation of gene transcription, which leads to significant changes in the repertoire of cellular proteins. This raises the question of how differentiation will affect the levels and activity of key proteins involved in base excision repair (BER) and nonhomologous end joining (NHEJ), the main DNA repair systems that remain active in post-mitotic neurons. In particular, the amounts and activities of PARPs in cells require reliable characterisation. DNA containing lipophilic substituents may be a useful tool for this purpose. One more interesting issue is how lipophilic residues in DNA influence the functional characteristics of DNAs, particularly the substrate properties in the 5′dRP lyase assays of some DNA repair proteins and the activating characteristics in autoPARylation catalysed by PARP1 and PARP2. To this end, we used several biochemical approaches to compare the binding of DNA duplexes with lipophilic groups (hereafter referred to as LS-DNAs) to PARP1, PARP2, PARP3, and DNA polymerase β and estimate their activity in removing 5′dRP groups at DS ends. In all cases, the introduction of lipophilic groups led to more efficient binding of LS-DNAs with the proteins above and proteins of the whole-cell extract (WCE). For the first time, we demonstrated the ability of DNA-dependent PARPs, PARP1, PARP2, and PARP3, to slowly remove 5′dRP groups from double-stranded DNA ends. The introduction of LS into DNA positively influenced the 5′dRP lyase activity of PARP2 and PARP3 at the DS ends, while the effect on the Ku antigen and PARP1 activity was less pronounced. The introduction of LS into 5′dRP DNA considerably increased the yield of DPCs with PARP3. The TZD group was more effective than the cholesterol substituent at improving DNA binding with the studied proteins.

In WCE 5′dRP-NHEJ, substrates formed DPCs with a considerable amount of protein targets, with DCPs of PARP1 and Ku antigen being clearly detectable. In the extracts, photoactivatable NHEJ substrates selectively modified the Ku antigen.

Lipophilic substituents significantly impaired the capability of LS-DNA to activate catalytic activity of PARP1 and PARP2. Under conditions of PAR synthesis catalysed by PARP1 and PARP2, the yield of DPCs with photoactivatable DNAs was considerably reduced.

## 2. Results and Discussion

### 2.1. Borohydride Trapping of PARPs

We previously showed the ability of PARP1 and PARP2 to excise the 5′dRP groups at the nicks flanked by 5′dRP and 3′OH in BER intermediates via β-elimination [21,41]. These BER intermediates appear as a product of AP site hydrolysis catalysed by APE1 [42]. In cells, the 5′dRP groups in BER intermediates are mainly removed by the 5′dRP lyase activity of pol β, which is known as the main 5′dRP lyase of this DNA repair pathway [42,43,44].

The aldehyde form of deoxyribose in the dRP group is potentially capable of reacting with the primary amino group of the protein in its vicinity to form a Schiff base. Therefore, the formation of this intermediate does not automatically imply that the protein has lyase activity; the presence of this activity has to be verified. In some cases, the Schiff-base-dependent intermediate may appear due to favourability for the reaction and mutual arrangement of the dRP group and the primary amino group of the protein, in particular the side amino group of lysine or the N-terminal amino group of the polypeptide chain. The transient Schiff-base-dependent covalent complex can be converted into an irreversible covalent adduct, that is, a DNA–protein cross-link (DPC), by treatment with sodium borohydride (NaBH_4_) or related compounds. In any case, the formation of such products clearly indicates direct contacts of specific regions of the DNA and the protein. This approach of DPC formation is sometimes referred to as borohydride trapping. The formation of cross-linking products and their analysis are shown schematically in Figure 1A.

The dRP lyase reaction proceeds from an enzyme–dRP–DNA complex to an enzyme–dRP complex (Figure 1A). Lower-electrophoretic-mobility products correspond to the protein with the DNA chain cross-linked (after Schiff base formation but before β-elimination). It should be noted that in the Laemmli method [51] of sample preparation for SDS-PAAG electrophoresis, no special compounds are added to melt the DNA duplexes. If the DNA duplex is not completely melted during sample preparation, an additional product with the lowest mobility may appear (not shown in the scheme). Products with a higher electrophoretic mobility correspond to the protein with the attached dRP group; these products appear after β-elimination, resulting in the release of the DNA chain. Note that these products are not detectable if the 5′dRP-bearing oligonucleotide contains a radioactive label at the 3′ end of the DNA chain.

**Figure 2:**
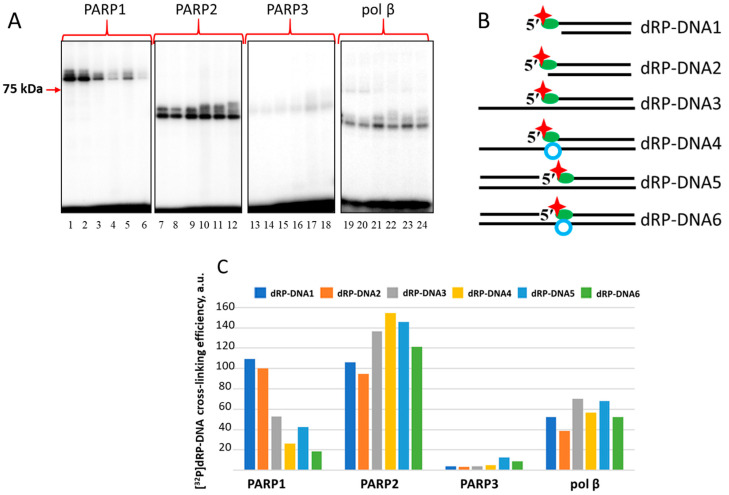
5′dRP-DNA cross-linking to PARP1, PARP2, PARP3, and pol β. Covalent cross-linking of proteins to 5′dRP DNAs was performed as described in the Materials and Methods, Section 3.2.6. Concentrations of reagents were 100 nM PARP1, 250 nM PARP2, 500 nM PARP3, 50 nM pol β, and 100 nM DNAs, time of the reaction was 10 min. (**A**)—Autoradiograph of the PAAG and (**B**)—schematic representation of dRP-DNAs. The positions of the substituents are designated as follows: the 5′dRP groups—green ovals, the radioactive label—red asterisks, AP sites—blue rings. (**C**)—Quantification of the products in the gel.

Given the abovementioned ability of PARP1 and PARP2 to remove 5′dRP groups in BER substrates via a β-elimination mechanism, we were curious to determine whether all three DNA-damage-dependent PARPs, PARP1, PARP2, and PARP3, are able to interact with and remove the 5′dRP residue in model DNA repair substrates of different structures, including DNA duplexes with lipophilic substituents. The data from the electrophoretic analysis of the purity of the proteins used in the study is shown in Figure S1. First, using regular oligonucleotides, we prepared 5′dRP-bearing DNAs of different structures, including NHEJ-mimicking substrates (dRP-DNA1–dRP-DNA6 in Table 1 and Figure 2B). The 5′dRP-DNAs contained ^32^P atom as part of the 5′dRP group.

For additional DNAs used in the study see Table S1.

5′dRP groups in DNA are metastable and were, therefore, generated by uracil removal with uracil DNA glycosylase immediately prior to the cross-linking experiments. Under the conditions used, approximately 95% of the uracils in all DNAs were removed.

Data on 5′dRP DNA cross-linking to proteins are shown in Figure 2. All proteins form several DPC products on PAAGE according to the Laemmli method [51].

Interestingly, PARP1 forms DPCs more efficiently with 5′dRP-DNA1 and 5′dRP-DNA2, mimetics of double-strand breaks (DSBs), than with 5′dRP-DNA5 and 5′dRP-DNA6, which can be considered as DNA intermediates of BER. 5′dRP-DNA6 represents a pattern of multiple damage sites where the DNA damage, the nick and the AP site, is located in opposite chains. PARP2 forms DPCs with all DNAs, with 5′dRP-DNA1 and 5′dRP-DNA2 being the least efficient in cross-linking among the DNAs tested. The low yield of DPCs formed by PARP3 may be a consequence of the low affinity of PARP3 to DNAs or the absence of Lys residue(s) at a position appropriate for interaction with the dRP residue. Unexpectedly, pol β demonstrates comparable levels of DPCs, irrespective of 5′dRP DNA structure. It has been previously reported that pol β inefficiently removes the 5′dRP groups located near DS ends [26,27,28]. 

### 2.2. PARP Binding with LS-DNAs as Revealed by EMSA

Having established the ability of PARPs and pol β to interact directly with DS breaks, we then investigated the influence of lipophilic substituents in NHEJ substrate DNAs on their binding to proteins and the functional properties of these DNAs.

The structures of the DNAs used for EMSA are shown schematically, as follows, in Figure 1B: DNA-R, DNA-TZD, DNA-PG-TZD, and DNA-Chol. The DNAs were composed of two chains, one of which was 30 nucleotides long and bore modification(s), as follows: the triazinylphosphoramidate group functionalised with two dodecyl substituents near the 3′ end (O-TZD, Table 1), a TZD group near the 3′ end and two phosphorylguanidine groups near the 5′ end (O-PG-TZD, Table 1), or a cholesterol residue attached to the 3′ end via a linker (O-Chol, Table 1). The control DNA-R was composed of two regular chains. In all DNAs, the complementary 26-nt chain (O1-Pho, Table 1) was labelled at the 5′ end with [^32^P] phosphate. These DNA duplexes with truncated chains were used to synthesise photoactivatable DNAs for the affinity labelling of proteins. Data on DNA binding by pol β at different concentrations are shown in Figure S2. Pol β binds LS-DNAs more efficiently than DNA composed of regular oligonucleotides (lanes 1, 6, 11, and 16) or regular oligonucleotides and oligonucleotides with the FAM group (lanes 5, 10, 15, and 20). Under the conditions used, the latter two DNAs formed detectable amounts of complexes only when the concentration of pol β exceeded that of the DNAs by a factor of 5–10 (Figure S2, compare lane 16 with lanes 1, 5, and 11 for regular DNA and lanes 15 and 2 with lanes 5 and 10 for FAM-containing DNA duplexes). When the concentration of pol β exceeded that of the LS-DNAs, complexes with a lower electrophoretic mobility were formed, which appeared to contain more than one molecule of the enzyme.

Figure S3A shows data on the electrophoretic mobilities of free DNAs (no protein added) and PARP1–DNA complexes run on the same gel (Figure S3A,B). The EMSA analysis data for the mobility of free DNA (Figure S3A, lanes 1–4) show that free LS-DNAs appeared ‘smeared’ on gels, even in the absence of proteins.

**Figure 3:**
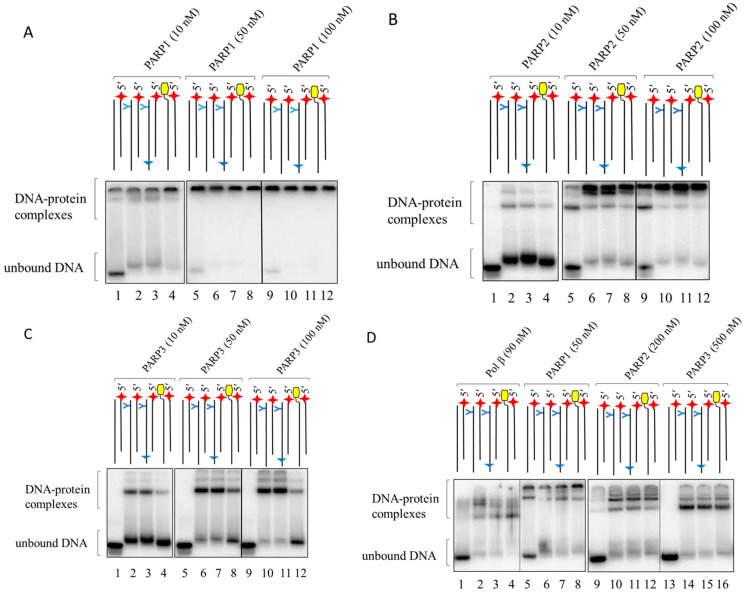
Binding of DNAs with PARPs as determined by EMSA. The EMSA was performed as described in the Material and Methods, Section 3.2.4. Concentration of DNAs was 10 nM (Panels (**A**–**C**)) or 100 nM (Panel (**D**)).

Analogous data on 10 nM DNAs’ binding to PARPs at different concentrations are shown in Figure 3A–C.

The data clearly demonstrate that PARPs more efficiently bound LS-DNAs as compared to DNA-R composed of regular oligonucleotides (compare lane 1 with lanes 2–4 in Figure 3A–C); under the conditions used, 10 nM PARPs and 10 nM DNAs, no complexes of regular DNA-R with PARP2 and PARP3 were detected, while about 30% of this DNA was bound by PARP1. PARP1 bound between 50% and 75% of LS-DNAs (Figure 3A, lanes 2–4). For PARP2, the maximal binding of LS-DNAs was 15% (Figure 3B, lane 2); for PARP3, this was 25% (Figure 3B, lane 2). An additional example of the protein binding with 100 nM DNAs is shown in Figure S4. 

In the case of PARP2 and PARP3, when the concentration of the protein exceeded that of the DNA, several types of complexes were clearly detected (Figure 3B,C). In the case of PARP1, such complexes did not appear to enter the gel and are visualised as a single band at the start position (Figure 3A, lanes 5–12). These lower-electrophoretic-mobility complexes appeared to contain more than one molecule of the protein per molecule of DNA. Given the Kd values of homodimeric complexes of PARP1 [52] or PARP2 [53] that are higher than 100 nM, these low-mobility complexes, which were detected even at 10 nM protein concentrations, hardly appeared due to the binding of DNA with the preformed dimeric form of PARPs.

In contrast to PARP1 and PARP2, PARP3 predominantly formed complexes of a higher electrophoretic mobility with a putative stoichiometry of 1:1, even at a 10-fold excess of PARP3 relative to DNA. PARP3, in contrast to PARP1 and PARP2, did not form detectable complexes with regular DNA, even at a 10-fold excess of the protein over DNA (lanes 1, 5, and 9 in Figure 3C). Taken together, these observations indicate a low affinity of PARP3 for regular DNA and a significant contribution of lipophilic substituents to ensure the formation of LS-DNA–PARP3 complexes.

Additionally, we used EMSA to test the binding of 100 nM DNA with proteins at the concentrations indicated in Figure 3D. These concentrations of DNAs and proteins will be used in subsequent experiments.

Having established an influence of LS on the binding of LS-DNAs to all DNA-dependent PARPs, we were curious to study how the introduction of LS groups interfered with the interaction of PARPs with regions of DNA duplexes near the DS ends. This can be achieved by the study of DNA–protein interaction using DNAs bearing chemically reactive groups near DS ends.

### 2.3. Probing of PARPs’ Interaction with Photoactivatable DNAs

One of the approaches used to study the interaction of proteins with DNA of different structures is affinity labelling, i.e., the cross-linking of the protein with DNA bearing chemically reactive groups capable of forming covalent complexes with proteins. The formation of a DPC indicates the direct interaction of the protein with a specific region of the DNA.

We used two types of DNAs. One type was DNAs carrying a 5′dRP group at the DS ends (dRP-DNA-R, dRP-DNA-TZD, dRP-DNA-PG-TZD, and dRP-DNA-Chol, Table 1 and Figure 1B), and the second type was photoactivatable DNAs containing the FAP-dCMP residue near the DS ends (Pho-DNA-R, Pho-DNA-TZD, Pho-DNA-PG-TZD, and Pho-DNA-Chol, Table 1 and Figure 1B). Notably, in the 5′dRP DNAs, the chemically reactive 5′dRP group was located near the LS (TZD or Chol) situated at the 3′ end of the complementary chain, whereas the photoactivatable FAP-dCMP group was located away from the LS, at another end of the DNA duplex. In addition, a photoreactive group was attached to the base via a rather flexible linker, while the 5′dRP group had no linker. Moreover, DNAs with 5′dRP located near their DS ends can be considered as potential substrates of specific 5′dRP lyase activity and, therefore, may be processed during incubation with the proteins under study. It should be noted that the yield of DPCs depends on the amount of preformed non-covalent protein–DNA complexes and the mutual orientation of the reactive group in DNA and a suitable amino acid target.

Photoactivatable DNAs can be synthesised in situ by pol β using FAP-dCTP as the substrate and then used for photo-inducible cross-linking experiments without DNA isolation. In pilot experiments under the optimal conditions, 87–89% of the primers were elongated by FAP-dCMP for all four DNAs. Data on the cross-linking of photoactivatable DNAs synthesised in situ are shown in Figure S5.

In order to avoid the potential influence of incomplete primer extension on the DNA cross-linking patterns, further experiments were performed on DNAs in which the FAP-dCMP residue was introduced into the oligonucleotide by pol β, followed by purification of the oligonucleotide by 7 M urea-PAAGE [54] and annealing of the purified oligonucleotide to the relevant complementary strand.

**Figure 4:**
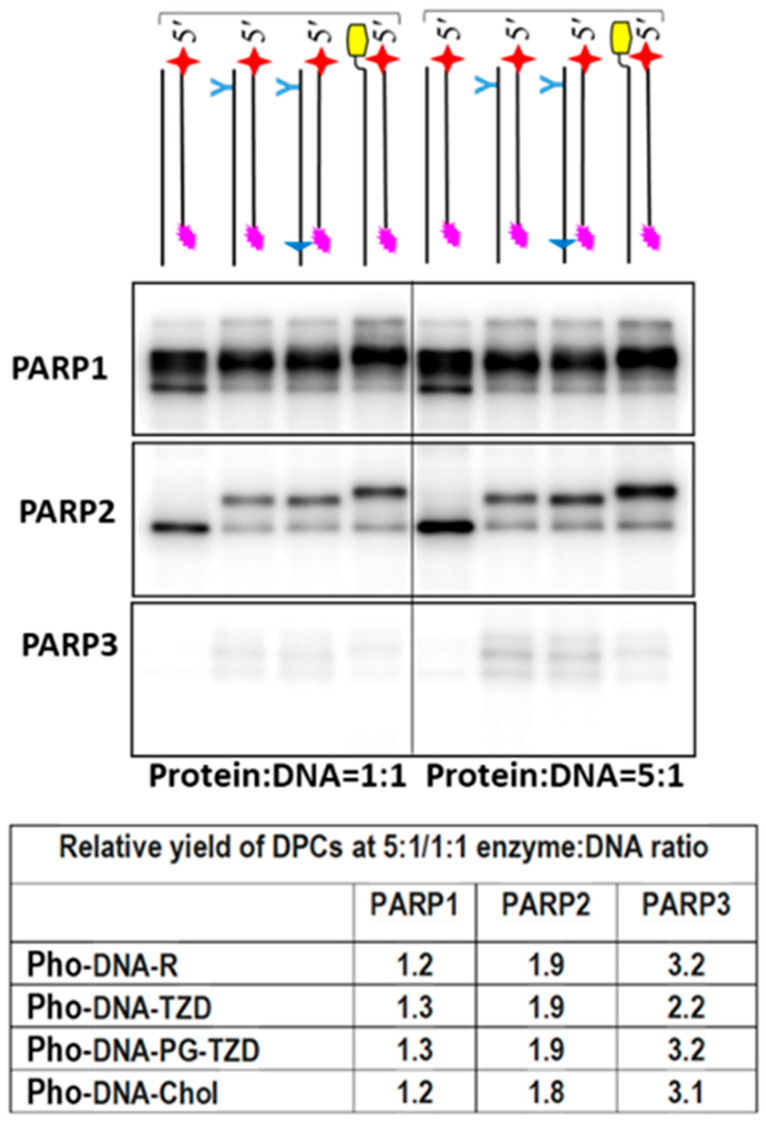
Affinity labelling of PARPs using photoactivatable NHEJ substrates. The concentrations of the reagents were as follows: 100 nM of photoactivatable [^32^P] DNA and 100 or 500 nM of PARPs. Cross-linking and other analyses were performed as described in the relevant section of Section 3. The products were separated using 12.5% SDS-PAAGE. The yield of DPCs with photoactivatable DNAs at a 1:1 enzyme-to-DNA ratio was taken as 1 for all PARPs.

Data on DPC formation and the relative yields of DPCs at a 5:1/1:1 enzyme–DNA ratio are shown in Figure 4. In the case of PARP1, which has a high affinity for DNA and, therefore, efficiently binds DNA even at an equimolar ratio, increasing the protein excess to five slightly increased the DPC yield by 1.2–1.3 times, while for PARP2, the DPC yield increased by approximately 1.9 times and for PARP3 by more than 3 times. These ratios are consistent with the expected change in the concentration of protein–DNA complexes with an increasing protein concentration.

Interestingly, Pho-DNA-R formed a few products of different electrophoretic mobilities with PARP1. These products may have arisen from the cross-linking of DNA to different amino acid targets due to the flexibility of the linker between the base and photoreactive group. In addition, such a product may be a consequence of melting of only part of DNA duplexes during sample preparation.

The cross-linking of a photoactivatable BER substrate (no LS in DNA) also revealed an extremely low amount of DPCs with PARP3 (Figure S6). Photoactivatable BER substrates contain an FAP-dCMP residue at the 3′end of the oligonucleotide, forming the nick.

Altogether, these patterns of photoactivatable DNA cross-linking are consistent with the lower affinity of PARP3 to DNA as compared to that of PARP1 and PARP2. In line with this finding is the low DCP formation with DNAs.

To further study LS influence on the efficiency of the protein interaction with DS breaks, we applied affinity modification of the proteins with DNA probes containing the 5′dRP group.

### 2.4. Probing of PARPs’ Interaction with 5′dRP DNAs

The 5′dRP-DNAs were composed of a regular chain containing [^32^P] 5′dRP groups and a complementary chain with LS (dRP-LS-DNA) or without a substituent for control DNA (Table 1 and Figure 1B, dRP-DNA-TZD, dRP-DNA-PG-TZD, dRP-DNA-Chol, and dRP-DNA-R, respectively).

**Figure 5:**
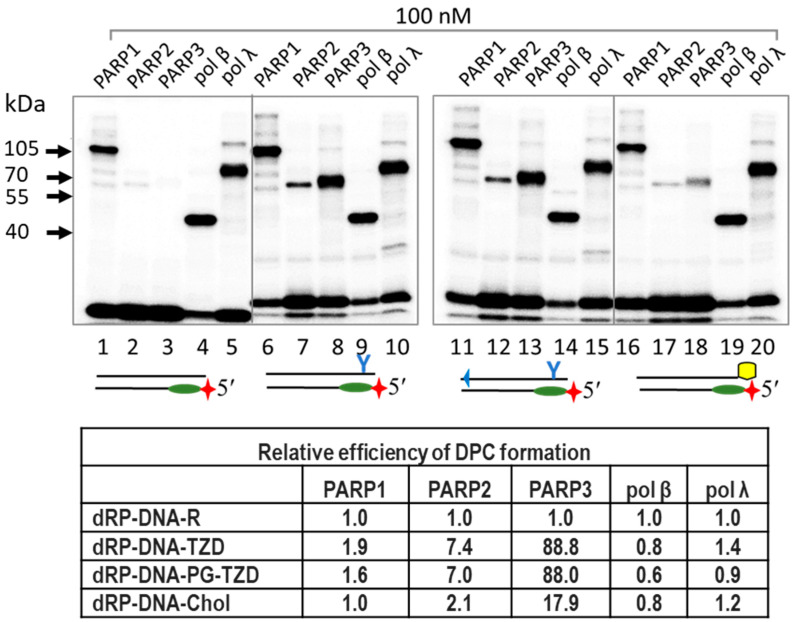
Formation of DPCs between 5′dRP-DNAs and DNA polymerases β and λ, and PARPs at equimolar ratio of DNA–protein. The reagent concentrations were as follows: 100 nM one of [^32^P]5′dRP-DNAs and 100 nM pol β, pol λ, PARP1, PARP2, or PARP3. The incubation time was 10 min. Cross-linking and other analyses were performed as described in the relevant section of Section 3. The products were separated using 12.5% SDS-PAGE. Relative efficiency is represented as the efficiency of DPCs for 5′dRP-DNA with LS normalised to that for regular 5′dRP-DNA-R.

Examples of DPC formation between PARPs, pol β, and pol λ for equimolar concentrations of DNA and proteins are shown in Figure 5 and Figure S7. All three PARPs were able to form DPCs with dRP-LS-DNA at least as efficiently as they could with the dRP-DNA. For each protein, the pattern of DPCs depended on the duration of protein incubation with DNA before borohydride treatment and the ratio of protein–DNA.

Pol β and pol λ very efficiently formed DPCs with all dRP-DNAs (Figure 5, lanes 4, 5, 9, 10, 14, 15, 19, and 20). The bottom panel shows the relative efficiency of DPC formation, estimated as the ratio of DPCs formed with dRP-LS-DNAs to those formed with dRP-DNA-R. It should be noted that for PARP2 and especially PARP3, the introduction of LS increased the efficiency of cross-linking compared to other tree proteins. This effect seemed to be due to more efficient PARP–DNA complex formation. Data on the relative efficiency of DPC formation for PARP3 at different ratios of PARP3–DNA are summarised in Table S2.

Pol β is known to be the major 5′dRP lyase in the BER process, removing 5′dRP at the nick, the intermediate formed as a result of APE1 activity [16,42,43,44], whereas poor 5′dRP lyase activity has been reported on NHEJ substrates (5′dRP is situated at DS breaks) compared to Ku antigen activity [26,27,28].

Unexpectedly, we found a rather efficient formation of DPCs between pol β and 5′dRP NHEJ DNA that indirectly testifies to efficient 5′dRP lyase activity at these substrates. The data represented in Figure S6 shows that at a 2 min reaction time with pol β and substrate DNA concentrations of 10 nM and 100 nM, respectively (multiple turnover conditions), DPCs are represented by two types of products with a comparable intensity, while at a nearly 1:1 ratio of pol β to DNA (90 nM pol β and 100 nM DNA), DPCs are mostly represented by products of a higher electrophoretic mobility, corresponding to adduct pol β-dRP. Such a pattern is consistent with a scenario where DNA chain cleavage has already occurred, but the sugar phosphate residue is still bound to the protein (see scheme in Figure 1). Pol λ, like pol β, exhibits 5′dRP lyase activity, consistent with a β-elimination mechanism [55]. Pol λ has been found to participate in microhomology-mediated end joining, the sub-pathway of Alt-NHEJ [56,57,58].

**Figure 6:**
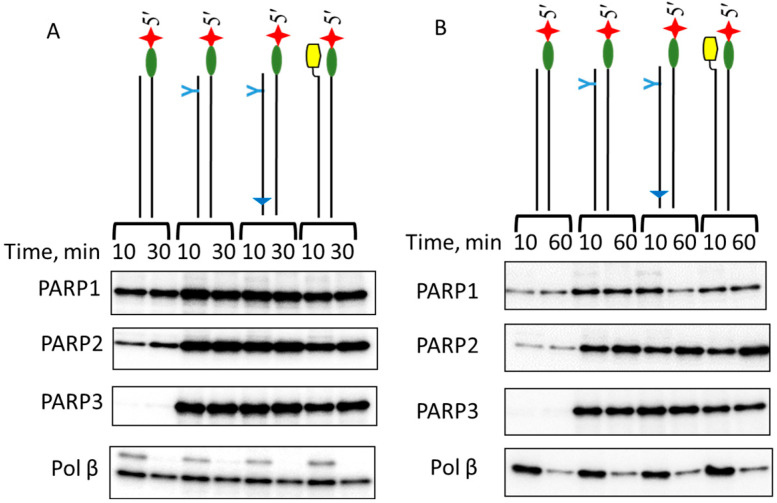
The kinetics of the decay of the transient intermediates formed by [^32^P]5′dRP-DNAs with one of the PARPs or pol β. The reagent concentrations were as follows: 100 nM [^32^P]5′dRP-DNAs, 100 nM PARP1, 500 nM PARP2, and PARP3. Pol β concentrations were as follows: 10 nM for panel (**A**) and 100 nM for panel (**B**). Cross-linking and other analyses were performed as described in the relevant section of Section 3. Products were separated by 10% SDS-PAAGE. Panel (**A**): times of the reaction were 10 and 30 min. Panel (**B**): times of the reaction were 10 and 60 min.

Data on the formation and decay of DCPs of PARPs and pol β with dRP-DNAs are shown in Figure 6.

The data clearly show that the rate of [^32^P]5′dRP-PARP adduct decay is low; even after a 60-min incubation period, cross-linking levels were reduced by less than 30%. The long persistence of the [^32^P]5′dRP-enzyme adduct has previously been reported for enzymes with slow 5′dRP lyase activity, such as polymerases γ and Klenow [15]. The difference observed between pol β cross-linking products at 10 min for Panel A and Panel B is due to the different pol β concentrations used, 10 nM and 100 nM, respectively. These conditions correspond to multiple and nearly single turnover conditions, and, therefore, the formation of new portions of [^32^P]5′dRP-pol β products generated in the case of low protein concentrations.

Having found DPCs of PARPs and pol β with 5′dRP-NHEJ substrates, characteristic products of 5′dRP lyase activity, we analysed lyase activity in another way that consisted of the detection of mobility shifts of a 30-mer 3′ end labelled oligonucleotide upon release of the 5′dRP group. The 5′ end positioning of the radioactive label does not allow for the detection of the oligonucleotide lacking the 5′dRP group, unlike the 3′ end positioning.

**Figure 7:**
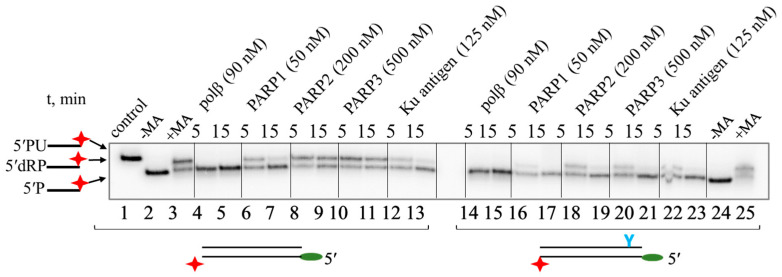
5′dRP lyase activity of pol β, PARP1, PARP2, PARP3, and Ku antigen on 5′dRP-containing DNAs. 5′dRP-oligonucleotide was labelled at the 3′ end via introduction of [^32^P]dCMP by activity of pol β as described in [21]. Concentrations of DNAs were 100 nM.

With the 3′ end labelling, both the substrate and the product of β-elimination carry the radioactive label. Analysis data are shown in Figure 7. All PARPs and the Ku antigen remove 5′dRP residue from the TZD-containing NHEJ substrate more efficiently than from the regular NHEJ substrate (Figure 7, compare lanes 6–13 with lanes 16–23). The Ku antigen was used here as a protein with proven 5′dRP lyase activity at the DS ends [26,27,28]. It has been shown earlier that pol β has one order of magnitude less efficient 5′dR lyase activity on the 5′dRP NHEJ substrate than on the 5′dRP BER substrate [26]. A more comprehensive study of pol β 5′dRP lyase activity reported that it is 60-fold more active on BER substrates than within a 5′ overhang of DSB substrates [45]. This pol β 5′dRP lyase activity is approximately 80-fold higher than the activity of DNA polymerase theta (Pol θ) [45] involved in TMEJ. We found that pol β (90 nM) at a concentration comparable to that of the Ku antigen (125 nM) removed 5′dRP residues much more efficiently than the Ku antigen, irrespective of the presence of LS in DNA duplexes. PARP1 (50 nM) and the Ku antigen (125 nM) showed a comparable 5′dR removal efficiency on both substrates tested. An additional example of the 5′dRP lyase activity of the same proteins is shown in Figure S8.

### 2.5. Probing of PARP Activation by LS DNAs

**Figure 8:**
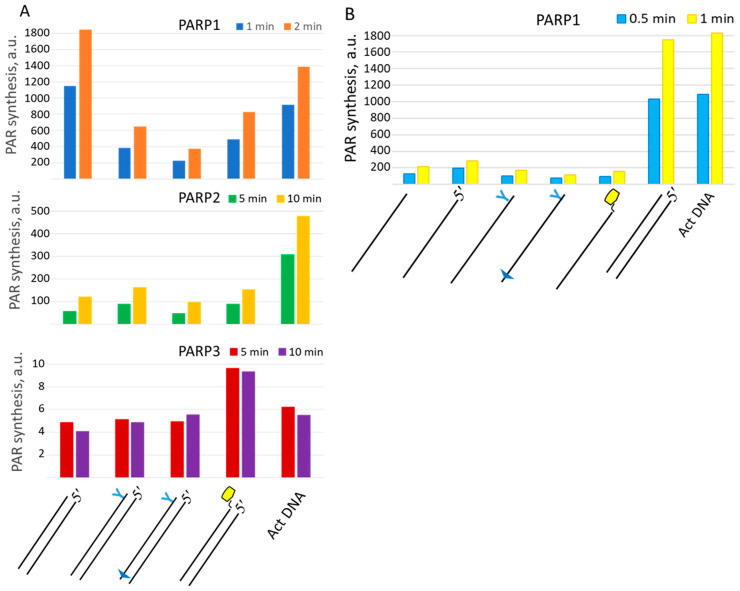
Activating properties of LS-DNAs in the reaction of auto-modification catalysed by PARP1–PARP3. The level of PAR synthesis was determined as described in the relevant section of Section 3. (**A**)—For DNA duplexes and (**B**)—for single-stranded DNAs, regular DNA duplexes and activated DNAs were used as controls. The concentrations of the reagents were as follows: 0.1 μM for DNAs, 0.6 A_260_/_mL_ for activated DNA, 50 nM for PARP1, and 500 nM for PARP2 and PARP3. The times of the reactions are shown at the top of the histograms.

To study how lipophilic groups interfere with the functionality of DNAs as cofactors of PARP activation, we tested the auto-modification of PARP1, PARP2, and PARP3 in the presence of different DNAs and [^32^P]NAD^+^ (Figure 8). In the case of PARP1, lipophilic groups in LS-DNAs decrease the activating efficiency by two to five times, depending on the DNA. DNA containing sugar–phosphate backbone modifications near both DS ends (TZD and PG) is the worst activator of PARP1 among those tested.

It should be noted that this DNA is the only DNA duplex in which the oligonucleotide contains a modifying group near the 5′ end. Introducing two phosphorylguanidine groups into the first and second internucleotide phosphates at the 5′ end neutralises the negative charge and appears to reduce this DNA’s ability to activate PARP1-catalysed PAR synthesis. PARP2 activation is less sensitive to the introduction of lipophilic groups into activating DNAs compared to PARP1. As expected, an extremely low incorporation of the radioactive label is observed for PARP3, which is consistent with its ability to transfer only one ADP-ribose moiety. Single-stranded oligonucleotides, irrespective of the presence of a modifying group, are poor activators of PARP1 (Figure 8B).

To check how the autoPARylation of PARP1 and PARP2 interfered with the binding of LS-containing DNAs, an examination of the cross-linking of PARP1 and PARP2 with photoactivatable DNAs under poly(ADP-ribosyl)ation conditions versus a control without NAD^+^ was performed. This approach has previously been used to study the influence of the autoPARylation of PARP1 and PARP2 on their binding with DNA [21,59,60].

**Figure 9:**
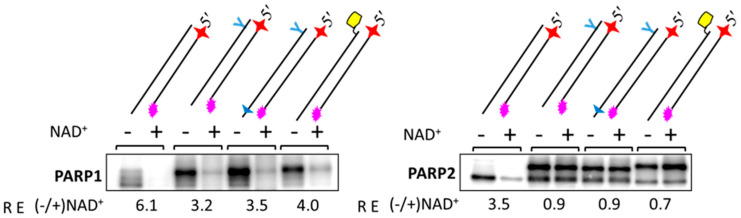
Influence of autoPARylation of PARP1 and PARP2 on their binding with DNA (cross-linking of PARPs with photoactivatable DNAs). PARP1 (200 nM) and PARP2 (200 nM) were incubated with the 100 nM 5′ ^32^P-labeled DNA probes containing FAPdCMP in the absence or presence of 400 mM NAD^+^ for 10 min at 37 °C. Cross-linking and other analyses were performed as described in the relevant section of Section 3. Products were separated by 10% SDS-PAGE. The RE (−/+ NAD^+^) values given below at the gel autoradiograph represent the ratio of the protein cross-links to DNA in the absence versus presence of NAD^+^.

PARPs were cross-linked to photoactivatable DNAs (Pho-DNA-R, Pho-DNA-TZD, Pho-DNA-PG-TZD, and Pho-DNA-Chol, Table 1) after incubation for 10 min at 37 °C in the absence or presence of NAD^+^ (Figure 9).

The relative efficiency, RE (−/+ NAD^+^), with values given below the gel autoradiograph, represents the ratio of the yield of DPCs in the absence versus presence of NAD^+^. In the presence of NAD^+^, the yield of PARP-Pho-DNA-R DPCs was significantly reduced for PARP1; a several-fold decrease in DPC levels with PARylated PARP1 was also observed for LS-containing DNAs. For PARP2, such a decrease was only observed for Pho-DNA-R, which does not contain LS. This scenario is consistent with the well-known more efficient autoPARylation of PARP1 compared to PARP2, which is due to the higher negative charge of the attached PAR bringing about the weakening of protein binding to DNA. For both PARPs, the more pronounced effect of autoPARylation in the case of DNAs without LS is consistent with the increase in protein affinity for DNA upon the introduction of LS.

### 2.6. Probing of Chemically Reactive DNA Interaction with Proteins in WCEs of HEK293, SH-SY5Y, and Olfactory Epithelial Cells

**Figure 10:**
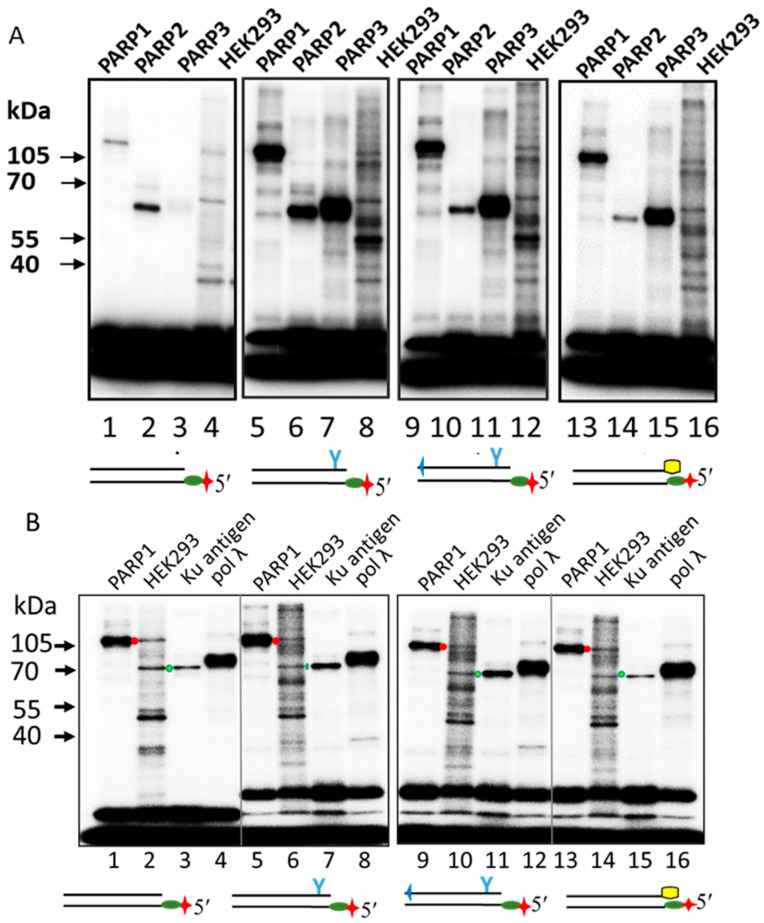
Comparison of cross-linking patterns of 5′dRP-DNAs with the purified proteins and proteins of WCEs. Reagent concentrations were 100 nM [^32^P]5′dRP-DNAs, 50 nM or 100 nM PARP1, 200 nM PARP2, 500 nM PARP3, 100 nM Ku antigen, 100 nM pol λ, and 0.75 or 1.5 mg/mL of HEK293 WCE for panel (**A**) and for panel (**B**), respectively. Cross-linking and other analyses were performed as described in the relevant section of Section 3. The products were resolved by 10% SDS-PAAGE for panel (**A**) and by 12.5% SDS-PAAGE for panel (**B**).

To detect the cellular proteins that interact with DNA double-strand breaks and determine how the introduction of LS into DNA affects this interaction, the same DNA probes were used to modify proteins in a whole-cell extract obtained from HEK293 cells. Data are shown in Figure 10A. Unexpectedly, a relatively high number of proteins formed Schiff-base-dependent cross-links with DNAs bearing the 5′dRP group at blunt DS ends. Again, overall, more DPCs were formed with 5′dRP-LS-DNAs, and the patterns of their cross-linking were rather similar, but significantly different from that observed for regular dRP-DNA-R. Apparently, not all proteins that formed DPCs with 5′dRP-containing DNAs had 5′dRP lyase activity. In some cases, such cross-linking may have occurred due to the accidental interaction of DNAs with proteins. DNA-recognizing regions of proteins are usually enriched in lysine residues and, therefore, are prone to DPC formation. The formation of a Schiff-base-dependent covalent DNA–protein adduct, accompanied by the slow release of the DNA chain, may be involved in the toxic effect of 5′dRP residues.

We and others have previously used DNAs of different structures with unexcised AP sites to search for proteins reactive to AP sites in cell extracts [12,17,60,61]. In those studies, a rather limited number of proteins were found to form detectable DPCs, and the target proteins depended on the structure of the AP DNA. In human WCEs, the Ku antigen and PARP1 were the preferred targets for a short linear abasic site containing DNAs [12,17,61,62]. It should be noted that the presence of targets detectable by the cross-linking technique depends on the affinity of the protein to DNA and the copy number of the protein in the extract. Both PARP1 and the Ku antigen have a high affinity for DNA, with Kd values for complexes being in the low nanomolar range [32,33,40,63,64,65]. Both proteins are very abundant in human cells. The amount of Ku antigen is estimated to be 500,000 molecules per cell, resulting in a nuclear concentration of 1.5 μM [32,33]. In HeLa cells, the copy number of PARP1 is estimated to be 2.4 million, giving a cellular concentration of 2 μM [66].

To test the ability of PARP1 and the Ku antigen to interact with 5′dRP-DNAs in the cell extract and the influence of LS on this interaction, we compare the patterns of DPCs in the extracts and those for purified PARP1 and Ku. In the extract samples, the presence of more intense radioactive bands with electrophoretic mobilities corresponding to those of the purified proteins allowed for indirect confirmation of the targets.

The overall efficiency of 100 nM 5′dRP DNA cross-linking to the proteins of HEK293 WCE was approximately 20–25% for regular dRP-DNA-R, 70–75% for dRP-DNA-PG-TZD, and 60–65% for dRP-DNA-Chol at a total extract protein concentration of 0.5 mg/mL. This result is consistent with a more efficient binding of LS-containing 5′dRP DNA to a large number of cellular proteins, making these DNAs and chemically reactive DNA probes based on them suitable tools for studying DNA–protein interactions.

High numbers of DPCs formed by extract proteins with electrophoretic mobilities corresponding to proteins with apparent molecular masses in the range of 50–70 kDa indicate the cross-linking of new unidentified targets (Figure 10). Some mammalian proteins, which possess a proven ability to form Schiff-base-dependent DPCs with 5′dRP DNAs, have molecular weights outside this range [18,19,22,24,25]. At the same time, PARP2, PARP3, and pol λ, with molecular weights in the range of 60–70 kDa, are able to form DPCs with 5′dRP DNAs, in particular with 5′dRP at DSB. Pol λ has been found to participate in microhomology-mediated end joining, the sub-pathway of Alt-NHEJ, and, therefore, its interaction with 5′dRP at DSBs is expectable [56,58]. A comparison of the electrophoretic mobility of DPCs of these pure recombinant proteins with that of cross-linked proteins of HEK293 WCE does not reveal any detectable bands whose electrophoretic mobility would be consistent with the electrophoretic mobility characteristic of DPCs of the above-purified proteins. The absence of detectable amounts of DPCs of PARP2 and PARP3 appears to be caused by the low copy number of these proteins in cells and, therefore, in whole-cell extracts compared to PARP1 and the Ku antigen that are detected in HEK293 WCE. Note that PARP1 and the Ku antigen, represented by high copy numbers in their cells and, therefore, in WCEs [32,33,66], form detectable DPCs with all 5′dRP-DNAs (Figure 10B, lanes 2, 6, 10, and 14). The bands corresponding to PARP1 and Ku antigen DPCs are marked on the autoradiographs by red and green dots, respectively (Figure 10B).

**Figure 11:**
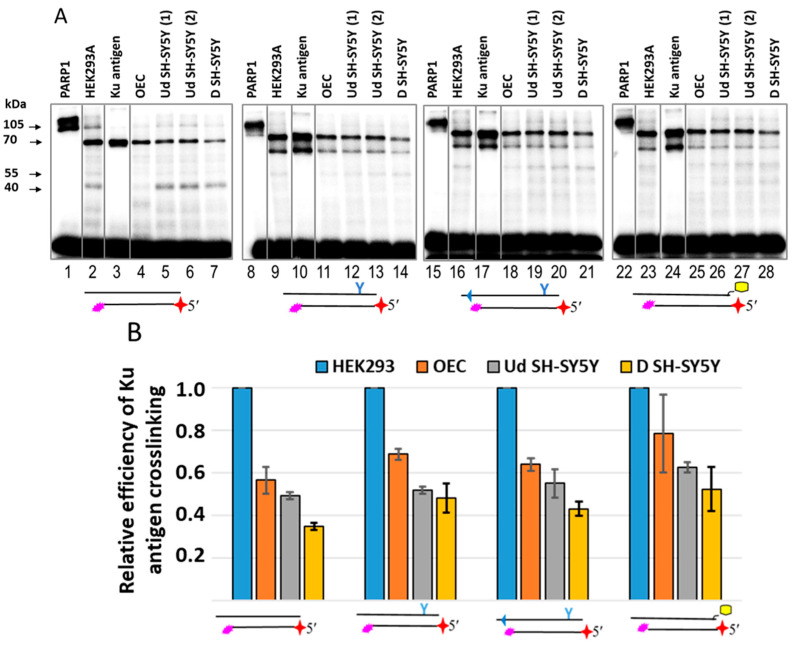
Comparison of the Ku antigen content in WCEs of HEK293 cells, undifferentiated SH-SY5Y cells (Ud SH-SY5Y), differentiated SH-SY5Y cells (D SH-SY5Y), parietal glial cells of the human olfactory epithelium (OEC), and individual PARP1 and Ku antigens, using photoactivatable NHEJ substrates. The reagent concentrations were as follows: 100 nM photoactivatable [^32^P]DNA, 50 nM PARP1, 125 nM Ku antigen, and 0.55 mg/mL WCE proteins. Cross-linking and other analyses were performed as described in the relevant section of Section 3. Panel (**A**) shows an example of Ku antigen–DNA cross-link analysis by 10% SDS-PAGE. Two samples of undifferentiated SH-SY5Y cells, designated UdSH-SY5Y (1) and UdSH-SY5Y (2), were used for this analysis. (**B**) Comparison of Ku antigen cross-linking in WCEs. The yield of DCPs was normalised to that in the HEK293 WCE. Data from three independent experiments are represented as the means ± SD.

To further study the LS influence on DNA interaction with the proteins in the extracts, we applied the cross-linking of photoactivatable analogues of NHEJ substrates (Pho-NHEJ DNAs), which is based on another mechanism of DPC formation. Data on the cross-linking of Pho-NHEJ DNAs are shown in Figure 11A and Figure S11.

A comparison of the DPC patterns obtained with the WCE proteins and individual PARP1 and Ku antigen DPCs allows us to classify the main protein targets in the extract as the Ku antigen. We have previously used the photoreactive DNA intermediates of the short and long patches of the BER process to detect proteins interacting with DNA in human cell extracts [6,8,17,62]. The DNA probes contained the photoactivatable nucleotide FAP-dCMP at the 3′ end of the oligonucleotide exposed to the nick, which were flanked at the 5′ end by 5′dRP (Pho-dRP) or 5′ pDEG (Pho-pDEG, where DEG is diethylene glycol). These photoactivatable DNAs formed DPCs of a comparable intensity with Ku70 and Ku80 and PARP1 at a 100 nM concentration of DNAs and PARP1 or the Ku antigen, or their mixture (Figure S6B, lanes 1–6). Both these proteins were predominant targets in HEK293 WCE (Figure S6B, lanes 7, 8).

An example of the cross-linking of two types of chemically reactive DNAs to the proteins of HEK293 WCE is shown in Figure S9.

The absence of DPCs corresponding to PARP1 in WCE in the case of Pho-NHEJ DNA may be related to significant differences in the affinity of PARP1 and Ku for DNA probes mimicking the substrate of BER (32 nt blunt end DNA duplex with nick) and NHEJ (30 nt DNA duplex without nick). Both proteins are characterised by a high affinity for single- and double-strand breaks, with Kd values in the low nanomolar range [32,33,63,64,65]. To achieve a nearly equal cross-linking of photoactivatable DNAs mimicking NHEJ substrates to both PARP1 and Ku, a 25–50 molar excess of PARP1 over Ku is required. Interestingly, the introduction of LS into Pho-NHEJ DNAs alters the target of cross-linking in such a way that Ku80 becomes the major point of DNA attachment, indicating a different mode of Ku antigen binding to DNA. Comparing the cross-linking patterns of Pho-NHEJ DNAs and dRP-NHEJ DNAs with the extract proteins, it can be concluded that the dRP-NHEJ DNAs reveal more protein targets interacting with DNAs. Meanwhile, DNA probes based on Pho-NHEJ DNA proved highly effective and selective in detecting the Ku antigen in cell extracts.

We have previously used the AP site containing DNA as an efficient and selective DNA probe for tracking the Ku antigen levels in WCEs of human cells, including several melanomas [62]. The intensities of the DPCs in the extracts were shown to correlate positively with the amount of Ku antigen estimated by dot blot analysis.

It should be noted that Western blot analysis or the estimation of protein content based on mRNA levels do not always unambiguously reflect the content of active forms of protein, in particular in the case of multi-subunit proteins. In the case of the Ku antigen, the heterodimerisation of Ku80 and Ku70 is essential for the efficient loading of the Ku antigen onto DNA ends [67]. The cross-linking of chemically reactive DNA probes reveals the forms of the protein that retain the ability to bind DNA. Thus, the affinity labelling of proteins in the context of complex systems using chemically reactive analogues of DNA repair substrates can be used as a powerful tool to estimate the content of proteins specifically interacting with DNA probes that represent the intermediates of DNA repair processes.

In vitro cultures of neuronal cells provide an efficient platform to characterise protein functionality and the molecular mechanisms underlying specific phenomena, in particular neurological disorders. SH-SY5Y cells can be differentiated from a neuroblast-like state into mature human neurons by a variety of different mechanisms, including the use of retinoic acid [49]. Differentiation is maintained by the specific modulation of gene transcription, leading to marked changes in cellular proteins. This effect raises the question of how differentiation interferes with the status of DNA repair machinery, in particular the expression of the key DNA repair proteins.

Using Pho-NHEJ DNAs, we compared the Ku antigen levels in WCEs from undifferentiated and retinoic-acid-differentiated SH-SY5Y cells, parietal glial cells of the human olfactory epithelium (OECs), and HEK293 cells. Figure 11 shows an example of Pho-NHEJ DNA cross-linking. The highest yield of DPCs corresponding to the Ku antigen was observed in the WCE of HEK293 cells. The relative yield of DPCs corresponding to the Ku antigen is represented in Figure 11B. For all DNAs, it was 20–40% higher in the HEK293 WCE as compared to the other WCEs, which can indirectly reflect the higher amount of Ku antigen in HEK293 cells.

### 2.7. Probing of Activation Properties of DNAs in WCEs

**Figure 12:**
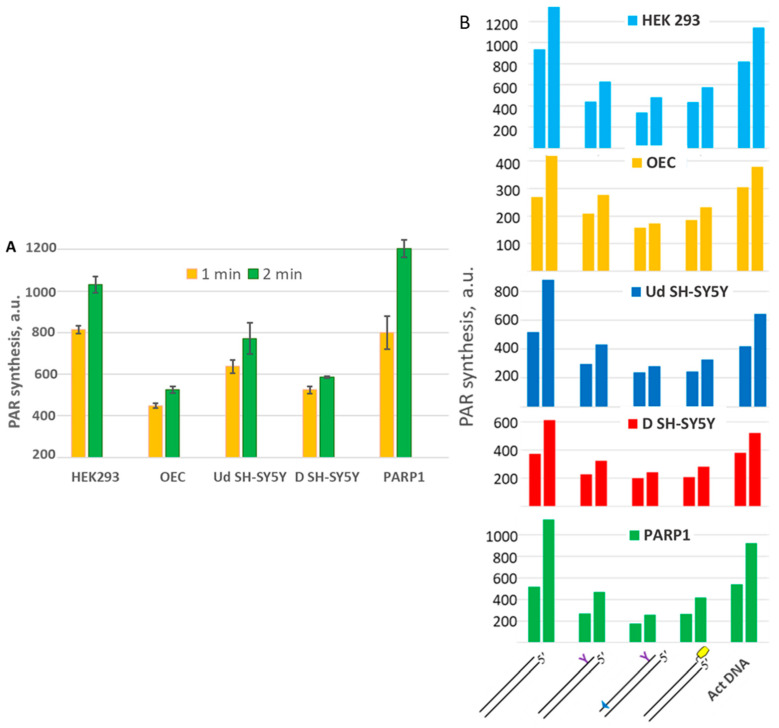
The activating properties of DNAs in PAR synthesis reactions catalysed by the endogenous PARPs of WCEs were investigated. The efficiency of PAR synthesis was estimated as described in ‘Activating properties of LS-DNA in the PAR synthesis’ (**A**). The concentrations of the main reagents were as follows: 0.25 mg/mL of WCE proteins; 7.5 nM PARP1; and 0.6 A_260_/mL of activated DNA. Reaction times were 1.0 and 2.0 min. Data are shown as the means ± SD of three independent experiments. (**B**). The concentrations of the main reagents were as follows: 0.25 mg/mL of WCE proteins, 7.5 nM PARP1, 0.6 A_260_/mL of activated DNA, and 0.1 μM of other DNAs. Reaction times were 0.5 and 1 min.

To further characterise the influence of LS on the activation properties of DNAs in the PAR synthesis reaction in WCEs, we first estimated the PAR synthesis levels by endogenous PARPs in the extracts, using activated DNA (DNAse-treated) as a cofactor (Figure 12A and Figure S10). The efficiency of PAR synthesis in WCEs followed the order of HEK293 > Ud SH-SY5Y > D SH-SY5Y > OEC. A similar pattern of PARP amounts was determined by WB analysis (Figure S12A). The qPCR data on the PARP1 mRNA levels in the WCEs shows the same order as the efficiency of PAR synthesis, but the difference between HEK293 and OECs and D SH-SY5Y is considerably higher (Figure S12B).

The activating properties of the DNAs under study in the PAR synthesis reaction catalysed by the endogenous PARPs of WCEs are shown in (Figure 12B). The profiles of PAR synthesis by the endogenous PARPs in WCEs for all DNAs, including activated DNA, resemble each other and recapitulate the profile observed for PARP1 (Figure 12 and Figure S13). This observation is in agreement with the main role of PARP1 in the PAR synthesis in cells. It should be noted that more than 90% of PAR in cells was synthesised by PARP1 [68]. The cellular proteins present in WCEs do not change the profile of PAR synthesis observed with recombinant PARP1.

Considering the use of short DNA duplexes to disrupt the DNA damage response in cells [4,10], the higher affinity of LS-containing DNAs for PARPs, coupled with their poorer ability to activate PARP1, leading to less efficient PAR synthesis and better DNA binding with PARP1, makes them more suitable than regular DNA duplexes.

## 3. Materials and Methods

### 3.1. Materials

TEMED, bis-acrylamide, MgCl_2_, Tris, SDS, DTT, bromophenol blue, NaBH_4_, bovine serum albumin, dithiothreitol, acrylamide, ammonium persulfate, EDTA, and glycine were obtained from Sigma-Aldrich (Burlington, MA, USA).

[γ-^32^P]ATP (5000 Ci/mmol) and [α-^32^P]ATP (3000 Ci/mmol) were produced in the Laboratory of Biotechnology, Institute of Chemical Biology and Fundamental Medicine SB RAS (ICBFM SB RAS, Russia, Novosibirsk). Exo-N-{2-[N-(4-azido-2,5-difluoro-3-chloropyridine-6-yl)-3-aminopropionyl]aminoethyl}-2′-deoxycitidine-5′-triphosphate (FAP-dCTP) was synthesised as described in [13].

The HEK293 cell line was from Thermo Fisher Scientific (Waltham, MA, USA). OECs (olfactory epithelial cells) were kindly donated by the Burdenko National Medical Research Center for Neurosurgery (Moscow, Russia). These OECs were derived from donor material and frozen at passages 3–5 for collection [69]. The cells were positive for the following markers: βIII-tubulin, which is a key protein of olfactory neurons; MAP2, which is an important structural component of dendrites; and GFAP (glial fibrillary acidic protein), which is specific to astrocytes and olfactory parietal cells. Cells at passages 9–12 were used for the experiments. SH-SY5Y cells were kindly donated by the Laboratory of Neurogenetics and Developmental Genetics of the Institute of Higher Nervous Activity and Neurophysiology of the Russian Academy of Sciences (Moscow, Russia). The supported laboratory stock was checked for the absence of mycoplasma contamination by PCR.

Plasmids bearing cDNA of rat pol β and human DNA polymerase λ (pol λ) were a kind gift from Dr. S.H. Wilson (National Institute of Environmental Health Sciences, Durham, NC, USA). The recombinant pol β was purified as described in [70]. The vectors coding for human PARP1 and PARP2 were a generous gift from Dr. V. Schreiber (École supérieure de biotechnologie de Strasbourg, Illkirch, France). The recombinant PARP1 and PARP2 were expressed in the insect cells and purified as described in [71]. A plasmid carrying a PARP3 cDNA was kindly provided by Dr. G. Zarkovich (Gustave Roussy, Universite Paris-Saclay, Paris, France). PARP3 was purified as described in [72]. Recombinant pol λ was a kind gift from Dr. E. Maltseva (Laboratory of Biotechnology, Institute of Chemical Biology and Fundamental Medicine SB RAS, Novosibirsk, Russia).

Human Ku protein was purified from a whole HEK293 cell extract by ammonium sulfate fractionation (45–65% of saturation), followed by successive chromatographies on DEAE Support (Bio-Rad, Berkeley, CA, USA), Q-Sepharose (GE Healthcare, Chicago, IL, USA), and DS-DNA-Cellulose (MP Biomedicals, Solon, OH, USA).

Polynucleotide kinases of T4 phage, *E. coli* UDG were purified from *E. coli* cells overexpressing the corresponding proteins.

Whole-cell extract (WCE) was prepared from HEK293, SH-SY5Y, and olfactory epithelial cells according to [73]. The protein concentration was determined according to [74].

Oligonucleotides and DNAs

Regular synthetic oligodeoxyribonucleotides (oligos) were obtained from the Laboratory of Biomedicinal Chemistry (Institute of Chemical Biology and Fundamental Medicine SB RAS, Novosibirsk, Russia). The oligos were 5′[^32^P]-labelled with T4 polynucleotide kinase and [γ-^32^P]ATP according to [54]. Unreacted [γ-^32^P]ATP was removed using a MicroSpin G-25 column (GE Healthcare, Chicago, IL, USA) according to the manufacturer’s protocol.

To obtain DNA duplexes, the complementary oligos were mixed in TE buffer (10 mM Tris-HCl pH 8.0 and 1 mM EDTA) at equimolar concentrations, followed by heating at 97 °C for 5 min and slow cooling down to room temperature. The resulting DNA duplexes were analysed by electrophoresis in a 10% PAAG under non-denaturing conditions. The sequences and structures of the DNAs used are shown in Table 1.

### 3.2. Methods

#### 3.2.1. Synthesis of [^32^P]NAD^+^

The synthesis of radioactive NAD^+^ from [α-^32^P]-ATP was carried out as previously described [75]. Briefly, reaction mixtures containing 1 mM ATP, 10 MBq of [α-^32^P]ATP, 20 mM MgCl_2_, 2 mM β-nicotinamide mononucleotide, and 5 mg/mL nicotinamide nucleotide adenylyl transferase in 25 mM Tris-HCl (pH 7.5) were incubated at 37 °C for 60 min. The reactions were heated at 90 °C for 3 min and denatured proteins were removed by centrifugation. The supernatant was used as a source of NAD^+^ without purification.

#### 3.2.2. Preparation of DNA Substrates Containing 5′dRP Residue

Due to the lability of the 5′dRP group, the uracil residues in the NHEJ substrates were removed by the activity of *E. coli* UDG immediately before the experiments. The reaction mixture consisted of 10 mM TE buffer pH 7.5, 1 μM uracil-containing DNA, and 0.1 U/μL UDG. The reaction was allowed to proceed at 37 °C for 20 min.

The oligonucleotide with dUMP at the 5′ end, which was used to prepare the 5′dRP lyase substrate, was radioactively labelled at the 3′ end via the introduction of dCMP using pol β and [α-^32^P]dCTP, after which it was purified using PAAGE.

#### 3.2.3. Activating Properties of LS-DNA in the PAR Synthesis

The reaction mixtures (10 μL) were composed of 0.6 A_260_/mL activated DNA or 100 nM NHEJ substrates (Table 1, DNA-R, DNA-TZD, DNA-PG-TZD, and DNA-Chol), 20 μM [^32^P]NAD^+^, 5 mM MgCl_2_, 50 mM Tris HCl (pH 8.0), 100 mM NaCl, 1 mM DTT, and 0.1 mg/mL BSA, which were mixed on ice. Then, 50 nM PARP1, 500 nM PARP2, 1 μM PARP3, or 0.55 mg/mL of WCE proteins was added, and the reaction mixtures were incubated at 37 °C for the specified time intervals. The reaction was stopped by applying 4 μL aliquots dropwise to Whatman 1 paper filters pre-impregnated with trichloroacetic acid (TCA). PAR bound to proteins was precipitated on the filters in the presence of TCA. To remove unreacted NAD^+^, the filters were dried and washed three times with 50 mL of 5% ice-cold TCA. The remainder of the TCA was removed from the paper with 90% ethanol, and the filters were dried and subjected to autoradiography for quantification.

#### 3.2.4. Electrophoretic Mobility Shift Assay (EMSA)

Electrophoretic analyses of the binding of oligonucleotide duplexes (Table 1, DNAs) with the proteins under study were performed using PAAG (8% PAAG, AA:BisAA = 40:1), 0.5 × TBE at 10 °C, 100 V. Protein–DNA binding was performed in a mixture of 50 mM Tris-HCl, pH 8.0, 20 mM NaCl, 0.25 mM EDTA, 5% glycerol, and 0.025% NP-40 and incubated at 37 °C for 20 min. After incubation, the reaction mixtures were supplemented with 2.5% Ficoll and the products were separated by PAAGE.

#### 3.2.5. Photoaffinity Modification of Proteins

The reaction mixtures for UV-inducible cross-linking (10 μL) contained 50 mM Tris-HCl (pH 8.0), 50 mM NaCl, 10 mM DTT, 15 mM EDTA, one photoactivatable DNA (Table 1, DNA) at 100 nM concentration, and 1 mg/mL cell extract proteins or one of the following purified recombinant proteins: 50 nM PARP1, 200 nM PARP2, or 500 nM PARP3, as indicated in the figure legend. The reaction mixtures were assembled on ice. Photolysis was induced by UV light using a Bio-Link-BLX cross-linker (VILBER-LOURMAT, Collégien, France) at 312 nm, 1.5 J/cm^2^ for 5 min on ice. After irradiation, the reaction mixtures were supplemented with Laemmli sample buffer and heated for 5 min at 97 °C, and the products were separated by 10% or 12.5% SDS-PAAGE [51] followed by autoradiographic analysis, as described below in the Section 3.2.10.

#### 3.2.6. Covalent Cross-Linking of Proteins to 5′dRP DNA

Reaction mixtures (10 μL) containing 50 mM Tris–HCl (pH 8.0), 50 mM NaCl, 10 mM EDTA, 0.1 μM [^32^P] dRP DNA, and one of the PARP1, PARP2, PARP3, Ku antigen, pol β, or pol λ proteins or WCEs of HEK293, SH-SY5Y, or OECs were mixed on ice and incubated for an indicated time interval at 37 °C. The concentrations of the proteins and times of the reaction interval are indicated in the figure legends. Then, NaBH_4_ was added to the reaction mixture to a final concentration of 20 mM and incubated for 30 min on ice. The reaction mixtures were supplemented with sample loading buffer, heated for 15 min at 90 °C, and the products were analysed by electrophoresis on 10% or 12.5% SDS-PAAG according to [51], followed by autoradiographic analysis, as described below in the Section 3.2.10.

#### 3.2.7. 5′dRP Lyase Activity Assay

Reaction mixtures (10 μL) contained 0.1 μM DNA duplex radiolabelled at the 3′ end of the dRP-containing oligonucleotide, one of the PARP1 (50 nM), PARP2 (0.2 μM), PARP3 (0.5 μM), Ku antigen (0.125 μM), or pol β (90 nM) proteins, and the following standard components: 50 mM Tris-HCl (pH 8.0), 50 mM NaCl, 10 mM EDTA, and 1 mM dithiothreitol. Samples were incubated and aliquots were taken after 0, 5 and 15 min. Control 5′dRP DNA was incubated for 15 min without added proteins and further treated as described below. The reaction was stopped by the addition of methoxyamine to a final concentration of 20 mM, followed by incubation at 0 °C for 30 min, and the products were analysed by 20% PAAGE in the presence of 7 M urea and 10% formamide according to [54].

#### 3.2.8. Cell Growth and Differentiation

HEK293 cells were grown in DMEM (Servicebio, Wuhan, China), 10% of foetal bovine serum (FBS), 100 U/mL penicillin–streptomycin, and 1 × GlutaMAX (Thermo Fisher Scientific, Waltham, MA, USA) at 37 °C with 5% CO_2_ in a humidified atmosphere.

OECs were cultured in alpha-MEM media (Sigma-Aldrich, Burlington, MA, USA) supplemented with 10% foetal bovine serum (Gibco BRL Co., Thermo Fisher Scientific, Waltham, MA, USA), GlutaMAX™ supplement (Sigma-Aldrich, Burlington, MA, USA), and 1 × Antibiotic-Antimicotic (Biowest, Nuaillé, France) in a humidified atmosphere of 5% CO_2_ at 37 °C.

SH-SY5Y cells were cultured in Dulbecco’s modified Eagle’s media (DMEM; Sigma-Aldrich) supplemented with 10% FBS, GlutaMAX™, and 1 × Antibiotic-Antimicotic in a humidified atmosphere of 5% CO_2_ at 37 °C.

For differentiation, SH-SY5Y cells were plated at a density of 2 × 10^5^ cells/T-57 flask in 12 mL of DMEM with 3% FBS, 1 × B27 supplement (ncB27, Nuwacell, Hefei, China), 1 × GlutaMAX, and 1 × Antibiotic-Antimicotic. The next day, a ½ volume of culture media was replaced with a portion of fresh culture media supplemented with retinoic acid (10 µM final concentration). Half of the medium was replaced every second day [76].

#### 3.2.9. Oligonucleotide Synthesis

The standard phosphoramidite solid-phase synthesis of all modified and unmodified oligonucleotides containing phosphodiester linkages was carried out on an ASM-800 DNA/RNA synthesizer (Biosset, Novosibirsk, Russia). Oligonucleotides were synthesised at the 0.4 μmol scale, using standard commercial 2-cyanoethyl deoxynucleoside phosphoramidites and CPG solid supports (Glen Research, San Diego, CA, USA). The insertion of a triazinyl phosphoramidate (TPA) modification bearing two dodecyl residues into appropriate oligonucleotide structures using a triazine modifier during the modified protocol of the oxidation step was performed as described in [6]. The insertion of a DMI modification into appropriate oligonucleotide structures during the modified oxidation step was performed using commercial 2-azido-1,3-dimethylimidazolidinium hexafluorophosphate (TCI, Tokyo, Japan) to obtain the 1,3-dimethylimidazolidin-2-ylidene phosphoramidate structure (DMI) as described in [7]. The synthesis of cholesterol-containing oligonucleotides was performed using modified CPG (Primetech, Minsk, Belarus) according to the manufacturer’s protocol. The introduction of 6-carboxyfluoresceine (FAM) was performed using the corresponding phosphoramidite (Lumiprobe, Moscow, Russia), as described in [8].

#### 3.2.10. Oligonucleotide Purification and Identification

Analytical RP HPLC was performed using a Millichrom A02 system equipped with a ProntoSIL-120-5-C18 column 2 × 75 mm (Econova, Novosibirsk, Russia) in a linear gradient of acetonitrile 0–50% or 0–90% in 20 mM triethylammonium acetate, pH 7.0, at a flow rate of 200 μL/min and with detection at 260, 280, and 300 nm wavelengths. Analysis of the derivatives bearing cholesterol moieties (H, HX) was performed through reverse-phase HPLC analysis on an Agilent 1200 HPLC system (Agilent, Santa Clara, CA, USA) using a Symmetry300 C4 5 μm column 4.6 × 150 mm (Waters, Milford, MA, USA) under the same conditions.

For oligonucleotide purification, RP HPLC was used on an Agilent 1200 HPLC system equipped with a Zorbax SB-C18 5 μm column 4.6 × 150 mm (Agilent, Santa Clara, CA, USA) or a Symmetry300 C4 5 μm column 4.6 × 150 mm (Waters, Milford, MA, USA) in a linear gradient of acetonitrile 0–50% or 0–90% in 20 mM triethylammonium acetate, pH 7.0, at a flow rate of 1.5 mL/min and with detection at 260, 280, 300, and 500 nm wavelengths. The desired fractions were collected and concentrated, and the oligonucleotides were precipitated with 2% LiClO_4_ in acetone. The precipitates were separated via centrifugation, washed with acetone, dried in air, and dissolved in deionised water.

#### 3.2.11. Quantification of the Results of Autoradiography

After the separation of the products, the PAAGs were dried and then examined by autoradiography. To this end, the gels were exposed with a radiosensitive screen, and radioactive products were visualised and quantified using a Typhoon FLA 9000 scanner (GE Healthcare, Chicago, IL, USA) and Quantity One software v. 4.6.6 (Bio-Rad, Berkeley, CA, USA).

## 4. Conclusions

We studied the influence of LS on the interaction of LS-DNAs with several enzymes involved in base excision repair and nonhomologous end joining. We found that, in all cases, introducing LS did not decrease DNA binding. In most cases, it even increased the efficiency of DNA binding with proteins, as shown by EMSA.

The most significant influence of LS was observed for PARP2 and PARP3, which exhibited inefficient binding to regular DNA duplexes, unlike LS-DNAs. In vitro, PARP2 and PARP3 demonstrated detectable 5′dRP lyase activity on the 5′dRP-DNA-TZD substrate, but not on regular 5′dRP-DNA-R substrates. Given that PARP2 and PARP3 have one and two orders of magnitude fewer copies, respectively, than PARP1, and given their low affinity for the regular 5′dRP NHEJ substrate, it is unlikely that they contribute significantly to the removal of 5′dRP from the DS ends of cellular DNA in cells with normal amounts of Ku antigen and PARP1. However, they may play a role in cells deficient in these proteins. Using [^32^P]5′dRP-DNA, we demonstrated the extremely slow release of the dRP moiety from the covalent complex with all three PARPs, rendering them inefficient 5′dRP lyases.

AsiDNA™, a DNA duplex with a special structure that bears a cholesterol group to facilitate penetration into cells, has been used to sensitise tumour cells to radiotherapy and chemotherapy [4,5]. Introducing a TZD group into DNA endows it with a range of promising properties as a DNA repair disturber, as follows:

(a) Increased efficiency in interacting with target proteins;
(b) Poor PARP1 activation, which leads to its persistence on these DNAs rather than binding to damaged cellular DNA;
(c) The ability to penetrate cells without transfectants [6,8];
(d) A very low cytotoxicity to cells in the absence of other impacts [6,8].

The functionalisation of DNAs with a TZD group could potentially provide an alternative way to impart the necessary characteristics to oligonucleotides for influencing DNA repair. In particular, these oligos could serve as a platform for developing therapeutics with targeted effects. Notably, the binding of TZD-containing DNAs to the studied proteins was more efficient than that of cholesterol-containing DNA. Additionally, this modification makes TZD-containing DNA a valuable tool for studying DNA–protein interactions.

## Figures and Tables

**Table 1:** DNAs used.

Oligos	Sequence	DNA Name
O-R	5′-TCCTGACATACTTGATACTTAGACATTCTT-3′	
O-TZD	5′-TCCTGACATACTTGATACTTAGACATTCT*T-3′	
O-PG-TZD	5′-T^x^C^x^CTGACATACTTGATACTTAGACATTCT*T-3′	
O-Chol	5′-TCCTGACATACTTGATACTTAGACATTCTT-[Chol]-3′	
U-O1	5′-UAAGAATGTCTAAGTATCAAGTATGTCAGGA-3′	
dRP-O1	5′-dRP-AAGAATGTCTAAGTATCAAGTATGTCAGGA-3′	
O1-Pho	5′-AAGAATGTCTAAGTATCAAGTATGT-3′	
O1-Pho-FAPdCMP	5′-AAGAATGTCTAAGTATCAAGTATGTC^φ^-3′	
O-R/O-R1	5′-TCCTGACATACTTGATACTTAGACATTCTT-3′ 3′-AGGTCAACTGTATGAACTATGAATCTGTAAGAA-5′	DNA-R
O-R/O-TZD	5′-TCCTGACATACTTGATACTTAGACATTCT*T-3′ 3′-AGGTCAACTGTATGAACTATGAATCTGTAAGAA-5′	DNA-TZD
O-R/O-PG-TZD	5′-T^x^C^x^CTGACATACTTGATACTTAGACATTCT*T-3′ 3′-AGGTCAACTGTATGAACTATGAATCTGTAAGAA-5′	DNA-PG-TZD
O-R/O-Chol	5′-TCCTGACATACTTGATACTTAGACATTCTT-[Chol]-3′ 3′-AGGTCAACTGTATGAACTATGAATCTGTAAGAA-5′	DNA-Chol
O-R/dRP-O1	5′-TCCTGACATACTTGATACTTAGACATTCTT-3′ 3′-AGGTCAACTGTATGAACTATGAATCTGTAAGAA-dRP-5′	dRP-DNA-R
O-TZD/dRP-O1	5′-TCCTGACATACTTGATACTTAGACATTCT*T-3′ 3′-AGGTCAACTGTATGAACTATGAATCTGTAAGAA-dRP-5′	dRP-DNA-TZD
O-PG-TZD/dRP-O1	5′-T^x^C^x^CTGACATACTTGATACTTAGACATTCT*T-3′ 3′-AGGTCAACTGTATGAACTATGAATCTGTAAGAA-dRP-5′	dRP-DNA-PG-TZD
O-Chol/dRP-O1	5′-TCCTGACATACTTGATACTTAGACATTCTT-[Chol]-3′ 3′-AGGTCAACTGTATGAACTATGAATCTGTAAGAA-dRP-5′	dRP-DNA-Chol
O-R/O1-Pho-FAPdCMP	5′-TCCTGACATACTTGATACTTAGACATTCTT-3′ 3′-C^φ^AACTGTATGAACTATGAATCTGTAAGAA-5′	Pho-DNA-R
O-TZD/O1-Pho-FAPdCMP	5′-TCCTGACATACTTGATACTTAGACATTCT*T-3′ 3′-C^φ^AACTGTATGAACTATGAATCTGTAAGAA-5′	Pho-DNA-TZD
O-PG-TZD/O1-Pho-FAPdCMP	5′-T^x^C^x^CTGACATACTTGATACTTAGACATTCT*T-3′ 3′-C^φ^AACTGTATGAACTATGAATCTGTAAGAA-5′	Pho-DNA-PG-TZD
O-Chol/O1-Pho-FAPdCMP	5′-TCCTGACATACTTGATACTTAGACATTCTT-[Chol]-3 3′-C^φ^AACTGTATGAACTATGAATCTGTAAGAA-5′	Pho-DNA-Chol
ds, 16/15	5′-dRPCCCGGCTTAGTCGCC-3′ 3′-GGCCGAATCAGCGG-5′	dRP-DNA1
ds, 16/16	5′-dRPCCCGGCTTAGTCGCC-3′ 3′-GGGCCGAATCAGCGG-5′	dRP-DNA2
ext/G, 16/32	5′-dRPCCCGGCTTAGTCGCC-3′ 3′-CCCTCCGGGACCGCAAGGGGCCGAATCAGCGG-5′	dRP-DNA3
ext/AP site, 16/32	5′-dRPCCCGGCTTAGTCGCC-3′ 3′-CCCTCCGGGACCGCAAOGGGCCGAATCAGCGG-5′	dRP-DNA4
nick/G, 16/32	5′-GGGAGGCCCTGGCGTT dRPCCCGGCTTAGTCGCC-3′ 3′-CCCTCCGGGACCGCAA GGGGCCGAATCAGCGG-5′	dRP-DNA5
nick/AP site, 16/32	5′-GGGAGGCCCTGGCGTT dRPCCCGGCTTAGTCGCC-3′ 3′-CCCTCCGGGACCGCAAOGGGCCGAATCAGCGG-5′	dRP-DNA6
x = PG	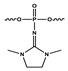 Phosphoryl guanidine modification	
* = TZD	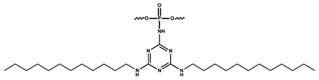 Triazinyl phosphoramidate modification with two dodecyl residues	
Chol = Cholesterol	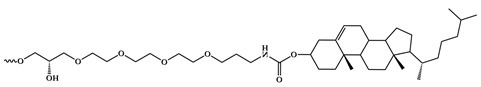 Cholesterol modification	
C^φ^ = FAPdCMP	Exo-N-{2-[N-(4-azido-2,5-difluoro-3-chloropyridine-6-yl)-3-aminopropionyl]aminoethyl}-2′-deoxycytidine-5′-monophosphate	
5′-dRP	5′-deoxyribosephosphate	
O	Apurinic/apyrimidinic site	

## Data Availability

The original contributions presented in this study are included in the article and Supplementary Materials.

## Supplementary Materials

The following materials are available online at https://www.mdpi.com/article/10.3390/ijms26157078/s1. References [77,78] are cited in the supplementary materials.

## Abbreviations

NHEJ	nonhomologous end joining
PAAGE	polyacrylamide gel electrophoresis
FAPdCMP	Exo-N-{2-[N-(4-azido-2,5-difluoro-3-chloropyridine-6-yl)-3-aminopropionyl]-aminoethyl}-2-deoxycytdine-5′-monophosphate
5′dRP	5′-deoxyribosephosphate
PARP1	poly(ADP)ribose polymerase 1
PARP2	poly(ADP)ribose polymerase 2
PARP3	poly(ADP)ribose polymerase 3
PAR	poly(ADP)ribose
PARylation	poly(ADP-ribosyl)ation
Pol β	DNA polymerase β
Pol λ	DNA polymerase λ
PG	phosphoryl guanidine
TZD	triazinylphosphoramidate group functionalised with two dodecyl substituents
OEC	human olfactory epithelium cells
