# Supplementary material for: Amphiphilic Oligonucleotide Derivatives as a Tool to Study DNA Repair Proteins
Source: Int J Mol Sci. 2025 Jul 23;26(15):7078. doi: 10.3390/ijms26157078 (PMC12346409; doi:10.3390/ijms26157078)
Supplement: Supplementary file 1 [file ijms-26-07078-s001.zip › ijms-3751848-supplementary.pdf]

SUPPORTING INFORMATION

# Amphiphilic oligonucleotide derivatives as a tool to study DNA repair proteins

Svetlana N. Khodyreva[1*], Alexandra A. Yamskikh[1,2,†], Ekaterina S. Ilina[1,2,†], Mikhail M. Kutuzov[1,3], Ekaterina A. Belousova[1,2], Maxim S. Kupryushkin[1], Timofey D. Zharkov[1], Olga A. Koval[1,2], Sophia P. Zvereva[1], Olga I. Lavrik [1,2,3*]

[1] *Institute of Chemical Biology and Fundamental Medicine, Siberian Branch of the Russian Academy of Sciences, 8 Akad. Lavrentyeva Ave., Novosibirsk 630090 Novosibirsk, Russia; svetakh@niboch.nsc.ru (S.N.K.), a.yamskikh@g.nsu.ru (A.A.Y.), katya.plekhanova@gmail.com (E.S.I.), kutuzov.mm@mail.ru (K.M.M.),* timazharkov74@gmail.com (T.D.Z.), kuprummax@niboch.nsc.ru (M.S.K.), zvereksonik@gmail.com (ZSP), olgaakoval@yandex.ru (K.O.A), *lavrik@niboch.nsc.ru (O.I.L),*

[2] *Novosibirsk State University, 2 Pirogova Str., Novosibirsk 630090 Novosibirsk, Russia; lavrik@niboch.nsc.ru (O.I.L.)*

[3] *Sechenov Institute of Evolutionary Physiology and Biochemistry, Russian Academy of Sciences, 44 Thorez pr., St. Petersburg 194223, Russia*

**Correspondence: svetakh@niboch.nsc.ru (S.N.K.); lavrik@niboch.nsc.ru (O.I.L.)*

*† These authors contributed equally to this paper*

## S.1. Supplementary Materials and Methods

### *S.1.1. Supplementary Materials*

Table S1. The oligonucleotide sequences used in this study

| DNA name | Sequences |
|---|---|
| O-R-FAM | 5'- FAM-TCCTGACATACTTGATACTTAGACATTCTT-3' |
| Pho-dRP | 5′-GGCGACTAAGCCGGGC$^{φ}$ d**RP**AACGCCAGGGCCTCCC-3′<br>3′-CCGCTGATTCGGCCCG TTGCGGTCCCGGAGGG-5′ |
| Pho-pDEG | 5′-GGCGACTAAGCCGGGC$^{φ}$ p**DEG**AACGCCAGGGCCTCCC-3′<br>3′-CCGCTGATTCGGCCCG TTGCGGTCCCGGAGGG-5′ |
| C$^{φ}$=FAPdCMP | exo-N-{2-[N-(4-azido-2,5-difluoro-3-chloropyridine-6-yl)-3-aminopropionyl]aminoethyl}-2′-deoxycytidine-5′-monophosphate |
| DEG | Diethylene glycol |

| | |
|---|---|
| FAM | 6-carboxyfluorescein |

S.1.2. Supplementary methods

*S.1.2.1. Preparing of Total RNA*

Total RNA was isolated using the TRIzol reagent (Thermo Fisher Scientific) according to ref. [77]. The purity of the isolated RNAs was accessed by means of the ratio of absorbance at 260 and 230 nm (A260/A230). A ratio of ~1.8–2.0 is generally assumed to indicate “purity” of RNA. For use in qPCR, the RNA was additionally treated with DNase to degrade possible traces of genomic DNA.

*S.1.2.2. qPCR Analysis of PARP1 mRNA Levels in HEK293, OEC and SH-SY5Y Cells*

To estimate relative expression of genes encoding PARP1 in HEK293, OEC, undifferentiated and differentiated SH-SY5Y cells reverse-transcription qPCR (RT-qPCR) analysis was performed. The reaction mixtures (20 µL) contained 0.5 ng/µL of total RNA, 0.5 µM primers, and 10 µL of BioMaster RT-qPCR SYBR Blue (2×). RT-qPCR was carried out on a LightCycler 96 system (Roche, Switzerland) under the following conditions: 1800 s reverse transcription at 45 °C, 300 s initial denaturation at 95 °C, and 30 cycles of 10 s 95 °C denaturation, 60 °C primer annealing, and 10 s 72 °C primer elongation. Fluorescence was recorded during the annealing/elongation step in each cycle. A melting curve analysis was performed at the end of each PCR by gradual increase the temperature from 58 to 95 °C with recording of the fluorescence. Signal detection was carried out at 84 °C for 5 s. A single peak in the melting temperature curve of the amplicons confirmed specificity of the primers. The RT-qPCR was carried out in triplicate. Housekeeping genes GAPDH, B2M. Primers were selected in the Primer-BLAST software (NCBI, USA). The sequences of forward/reverse primers for RT-qPCR were the following: *Gapdh (5′-* AGATCATCAGCAATGCCTCCT-3′/5′-TGGTCATGAGTCCTTCCACG-3′), *B2M (5′-* CGCTCCGTGGCCTTAGCTGT-3′/5′-AAAGACAAGTCTGAATGCTC-3′), *Parp1 (5′-* TGCCTATTACTGCACTGGGG-3′/5′-TCTCGGAATTCCTTTGGGGTT-3′). For each pair of primers, the amplification efficiency was found to be in the range of 90–110%.

*S.1.2.3. Western-Blot Analysis of PARP1 in WCEs of HEK293, OEC and SH-SY5Y Cells*

In brief, WCE proteins (2.5 µg) or PARP1 (0.05 µg) were resolved by 12.5% SDS-PAGE electrophoresis [51] followed by electrotransfer of proteins onto a nitrocellulose membrane using Trans-Blot Turbo (Bio-Rad, Hercules, CA, USA). The membrane was incubated in a solution of primary antibodies (rabbit antibodies to PARP1 at a dilution of 1:1000), then in a solution of

secondary antibodies conjugated to HRP. The conjugate was stained using Super Signal West Pico PLUS (Thermo Fisher Scientific, Waltham, MA, USA). Chemiluminescence was detected on Amersham Imager 600 (GE Healthcare, USA Chicago, IL, USA).

### S.2.1. Supplementary Results

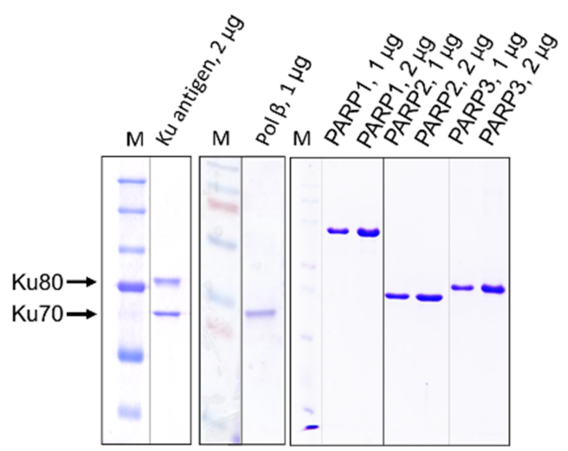


Figure S1. Electrophoretic analysis of proteins used in the study. The proteins were run on the 10% PAAG-SDS gel according to Laemmli method [51] and stained by colloidal Coomassie R-250 as described [78].

Binding of DNAs with pol β as determined by EMSA (Figure S2). LS in DNA considerably enhances binding of DNAs with pol β and at enzyme: DNA ratio of 10 all LS containing DNAs are bound while only slightly detectable amounts of complexes with regular DNA are observed (Figure S2 lanes 16 and 20 versus lanes 17-19).

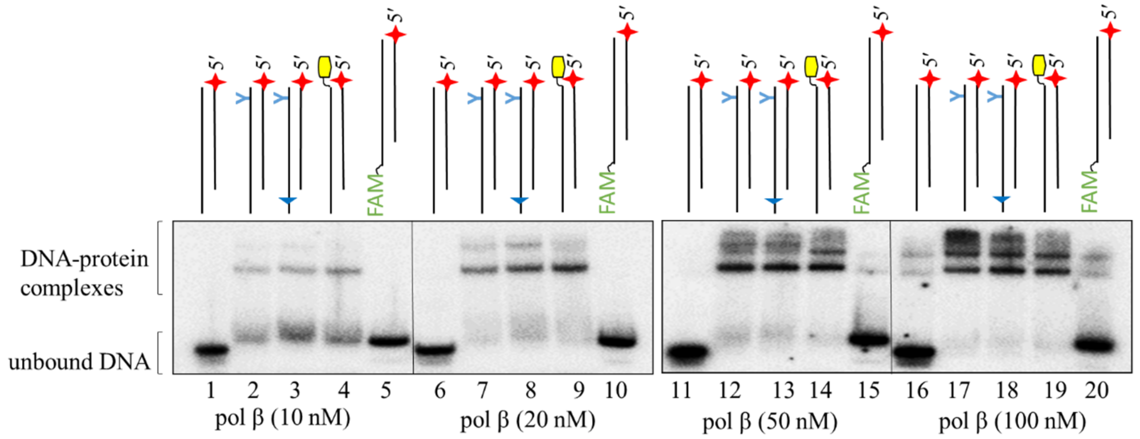


Figure S2. Binding of DNAs with pol β as determined by EMSA. The EMSA was done according to the section 3.2.4 of the Materials and Methods. Concentration of [$^{32}$P]DNAs was 10 nM.

Interestingly, for PARP3, in contrast to PARP1 and PARP2, no complexes with FAM-containing regular DNA were detected under the conditions used (10 nM DNA and 10 nM PARP1, PARP2

or PARP3) (Figure S3B, compare lanes 3 and 6 with lane 9). This difference should be taken into account when planning to use FAM-containing DNAs.

account when planning to use FAM-containing DNAs.

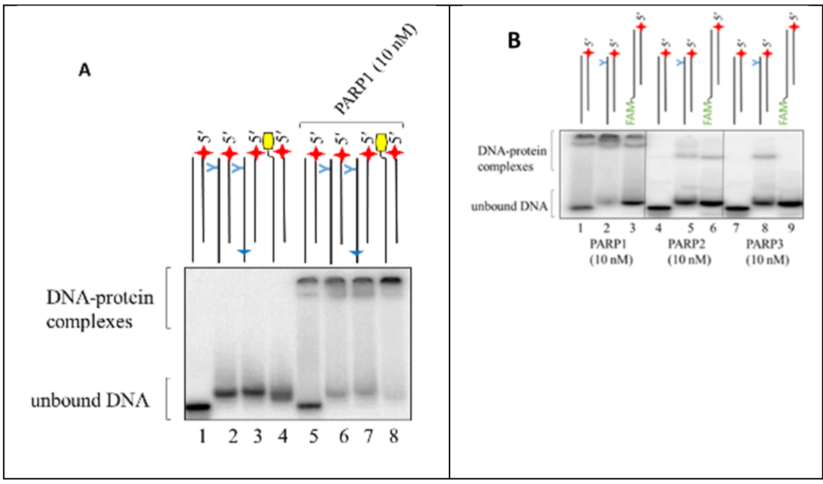


Figure S3.
A and B - Binding of DNAs with PARPs as determined by EMSA. The EMSA was done as described in the section 3.2.4. The concentration of DNAs was 10 nM.

Binding of DNAs with PARPs in the presence of pol β as determined by EMSA (Figure S4).

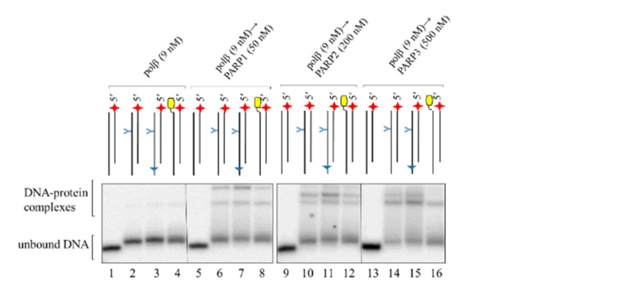


Figure S4.
Binding of DNAs with proteins as determined by EMSA. The EMSA was done as described in the section 3.2.3 of Materials and Methods. The concentration of DNAs was 100 nM.

Photoactivatable DNAs can be synthesized in situ by pol β using FAP-dCTP as the substrate and then used for photo-inducible crosslinking experiments without DNA isolation. In pilot experiments under the optimal conditions 87-89% of the primers were elongated by FAP-dCMP in all four DNAs. In this setting, the reaction mixtures for UV-inducible cross-linking will contain some pol β. Data on cross-linking of photoactivatable DNAs synthesized *in situ* are shown in Figure S5. PARP1 and PARP2 form DPCs quite efficiently, in contrast to PARP3.

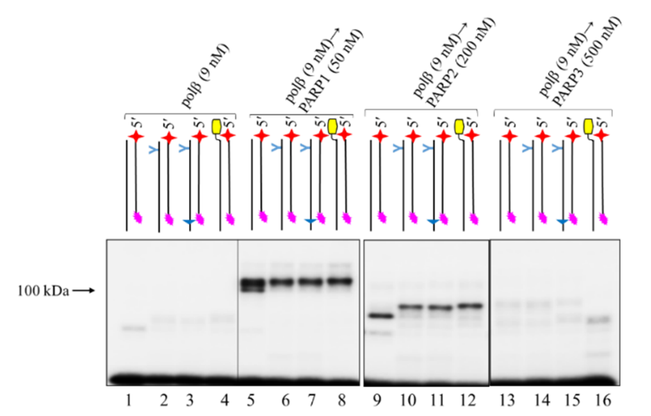


Figure S5. Affinity labelling of PARPs using photoactivatable NHEJ substrates synthesised *in situ*.
The reagent concentrations were: 100 nM photoactivatable [$^{32}$P] DNAs, and proteins concentrations as designated in the Figure. Cross-linking and other analyses were performed as described in the relevant section of the Materials and Methods. Products were separated 10% SDS-PAGE.

Cross-linking of photoactivatable BER substrate (no LS in DNA) revealed also extremely low amount of DPCs with PARP3 (Figure S6). Photoactivatable BER substrates contain FAP-dCMP residue at the 3′end of oligonucleotide forming the nick.

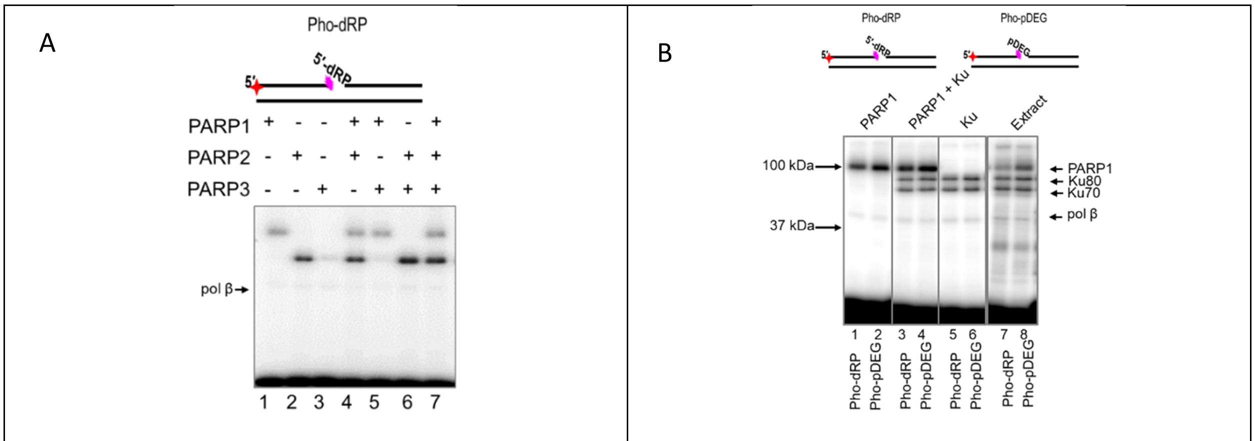


Figure S6. Affinity labelling of PARP1, PARP2, PARP3, Ku antigen and proteins of HEK293 WCE by photoactivatable BER substrate. Affinity modification of proteins by photoactivatable BER substrates were done as described in the 3.2.5 section. The concentrations of the reagents were 100 nM photoactivatable [$^{32}$P] DNAs, 100 nM PARP1, 200 nM PARP2, 300 nM PARP3 and 1 mg/mL HEK293 WCE.

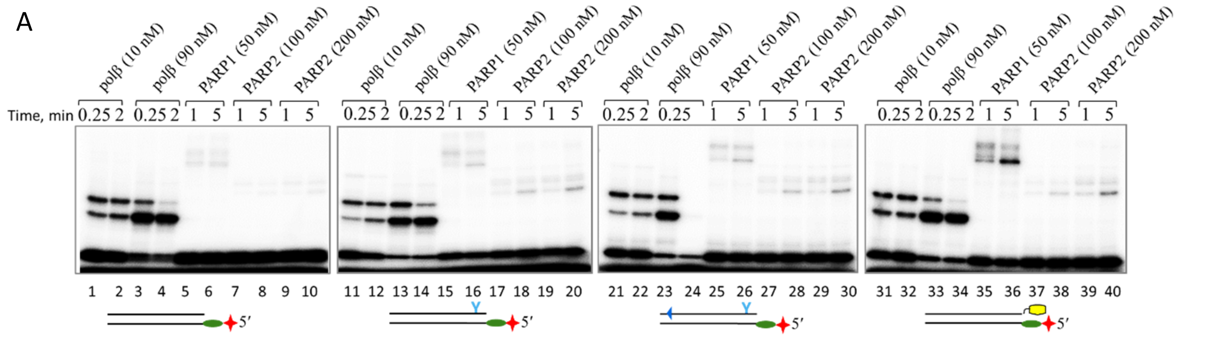

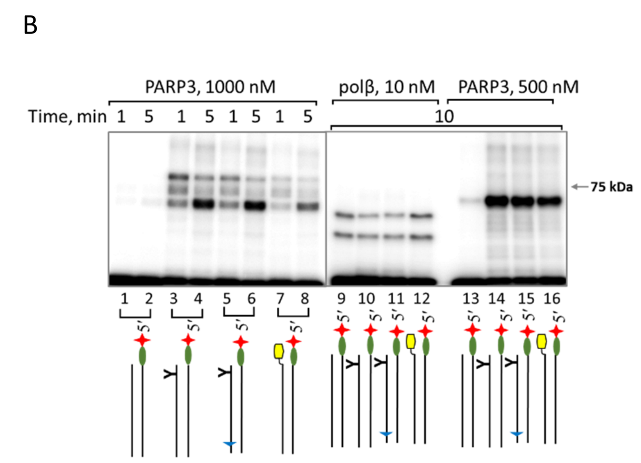


Figure S7. Formation of DPCs between 5′dRP-DNAs and DNA polymerases β and PARPs. Covalent cross-linking of proteins to 5′dRP DNAs was performed as described in the Materials and Methods, section 3.2.6. [$^{32}$P]5′dRP DNAs 100 nM, pol β 10 nM or 90 nM, PARP1 50 nM, PARP2 100 nM or 200 nM, PARP3 - 500 nM. The products were separated by 10% SDS-PAGE according to Laemmli method [51] and analysed as described in the Materials and Methods.

The higher proportion of pol β-dRP-DNA product over pol β-dRP-DNA product observed at 10 nM pol β compared to the ratio observed for 90 nM pol β is likely due to the fact that in the former case the reaction conditions correspond to a multi-turnover regime, causing the formation of new portions of pol β-dRP-DNA intermediate (Figure S7, lanes 1-4, 11-14, 21-24 and 31-34).

Table S2. Relative efficiency of DPC formation by PARP3 at different concentrations of PARP3

| | PARP3-0.1 µM<br>DNAs-0.1 µM<br>10 min | PARP3-0.5 µM<br>DNAs-0.1 µM<br>10 min | PARP3-1.0 µM<br>DNAs-0.1 µM<br>10 min |
|---|---|---|---|
| dRP-DNA-R | 1.0 | 1.0 | 1.0 |
| dRP-DNA-TZD | 88.8 | 42.5 | 22.9 |
| dRP-DNA-PG-TZD | 88.0 | 51.2 | 20.2 |
| dRP-DNA-Chol | 17.9 | 30.0 | 14.8 |

An example of dRP lyase activity of purified proteins on the 3’ labelled substrate is shown in Figure S8.

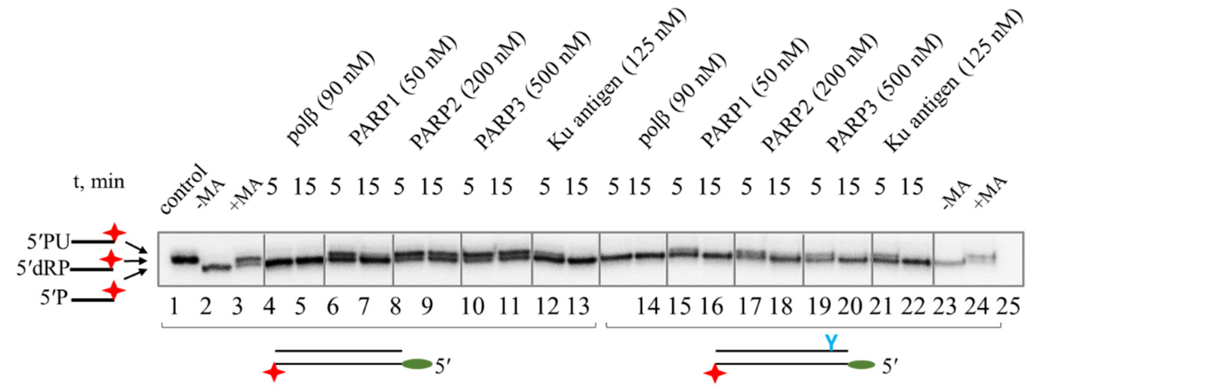

Figure S8. 5′dRP lyase activity of pol β, PARP1, PARP2, PARP3, and Ku antigen on dRP-containing DNAs. The 5′dU oligonucleotide was labelled at its 3′ end by introducing [$^{32}$P]dCMP via DNA polymerase β, which was complementary to the template dGMP. This was followed by purification of the oligonucleotide, annealing to the appropriate chain, and removal of the uracil residues.

An example of cross-linking of two types of chemically reactive DNAs to the proteins of HEK293 WCE is shown in Figure S9 demonstrates very selective labelling of the proteins in HEK293 by photoactivatable DNAs (Panel A) as compared to 5′dRP NHEJ substrates (Panel B).

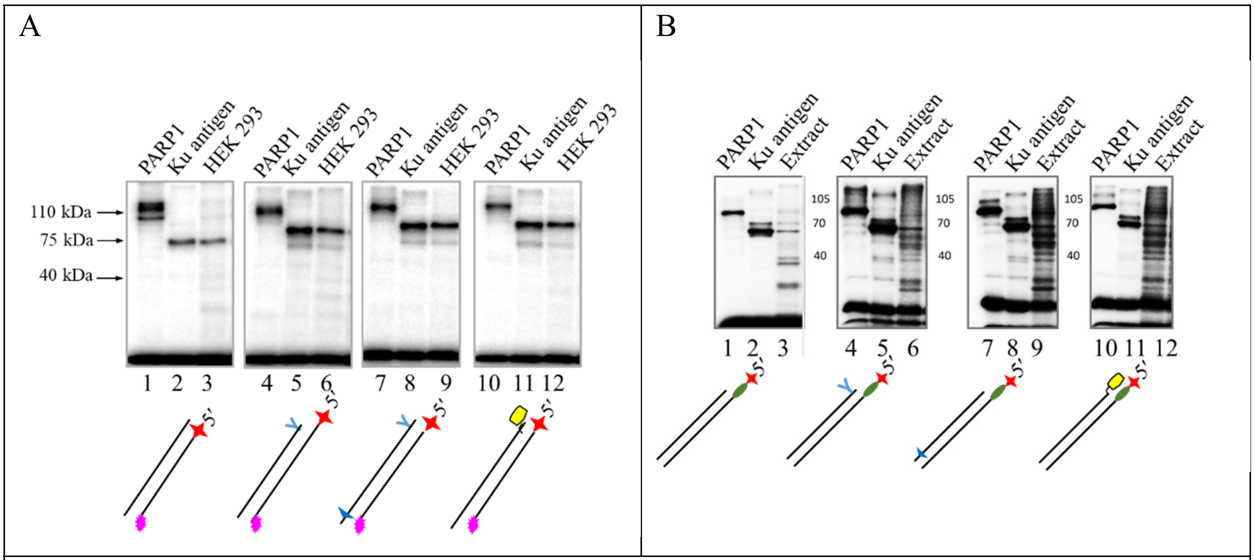


Figure S9. Affinity labelling of proteins in HEK293A WCE and individual proteins, PARP1 and Ku antigen, by photoactivatable (A) and 5′dRP NHEJ substrates (B). The reagent concentrations were 100 nM photoactivatable [$^{32}$P] DNAs, 100 nM PARP1, 125 nM Ku antigen and 1 mg/ml HEK293A WCE. Cross-linking and other analyses were performed as described in the corresponding section of the Materials and Methods. Products were separated by SDS-PAAGE, 10% for Panel A and 12.5% for Panel B.

An example of PAR synthesis by endogenous PARPs of WCEs (autoradiograph).

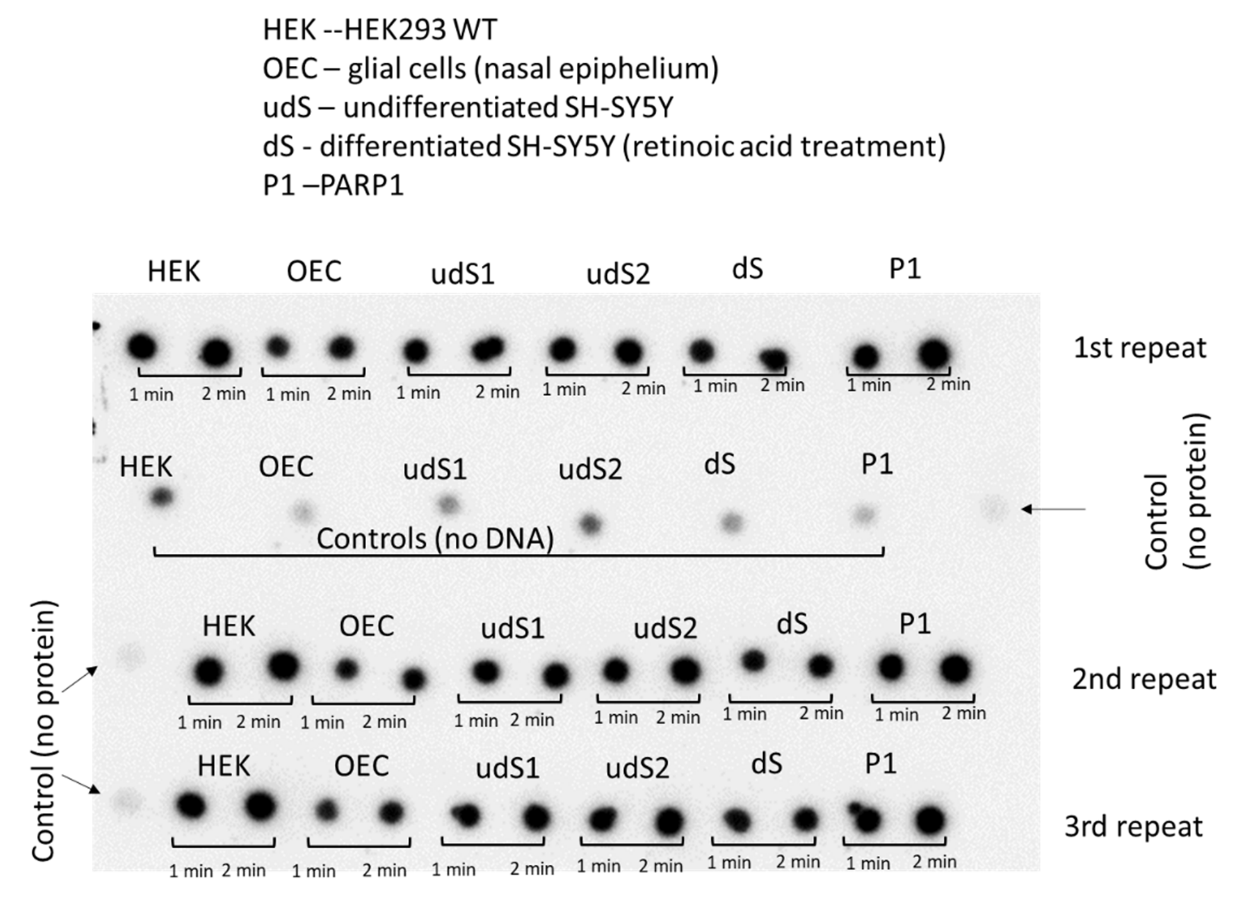


Figure S10. Synthesis of PAR by endogenous PARPs in the extracts. Activated DNA (0.6 $A_{260}$/ml) was used as a cofactor. Concentration of PARP 1 was 7.5 nM, concentration of the extract proteins was 0.55 mg/ml and concentration of [$^{32}$P]NAD$^+$ was 20 µM.

An example of affinity labelling of proteins in WCEs of HEK293, undifferentiated SH-SY5Y (Ud SH-SY5Y), differentiated SH-SY5Y (D SH-SY5Y), parietal glial cells of the human olfactory epithelium (OEC) and individual PARP1 and Ku antigen using photoactivatable NHEJ substrates is shown in Figure S11.

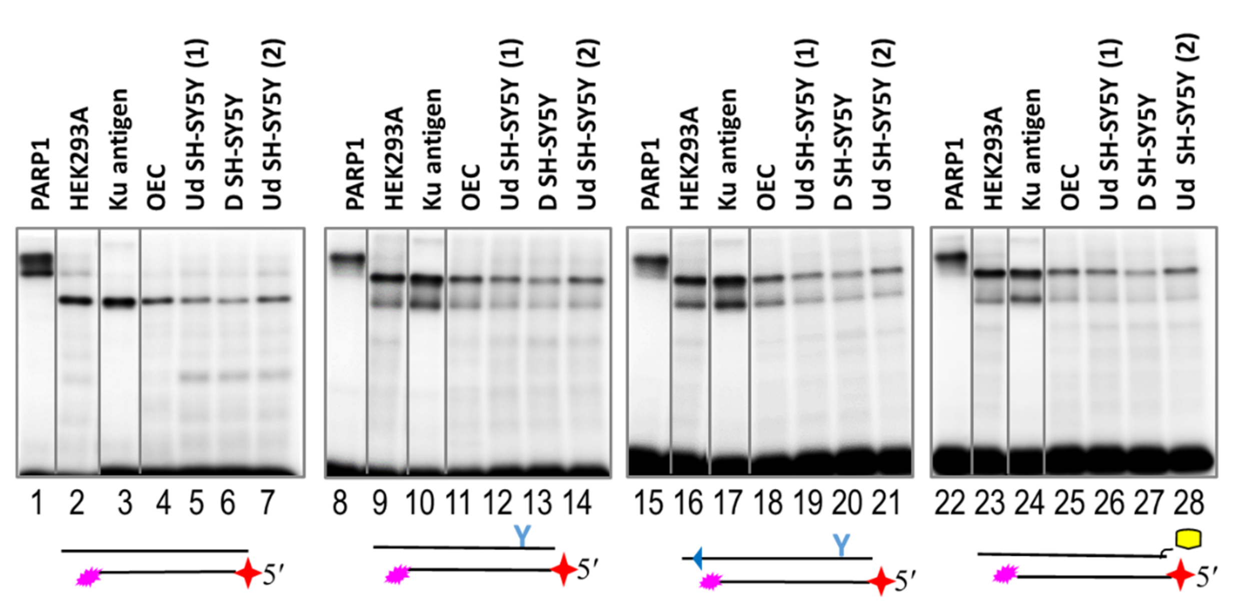


Figure S11. Affinity labelling of proteins in WCEs of HEK293, undifferentiated SH-SY5Y (Ud SH-SY5Y), differentiated SH-SY5Y (D SH-SY5Y), parietal glial cells of the human olfactory epithelium cells (OEC) and individual PARP1 and Ku antigen using photoactivatable NHEJ substrates.

Reagent concentrations of were as follows: 100 nM photoactivatable [$^{32}$P] DNA, 50 nM PARP1, 125 nM Ku antigen and 0.55 mg/ml WCE proteins. Cross-linking and other analyses were performed as described in the relevant section of the Materials and Methods. Products were separated by 10% SDS-PAGE. Two independently prepared samples of undifferentiated SH-SY5Y WCE designated by UdSH-SY5Y (1) and UdSH-SY5Y (2), were used.

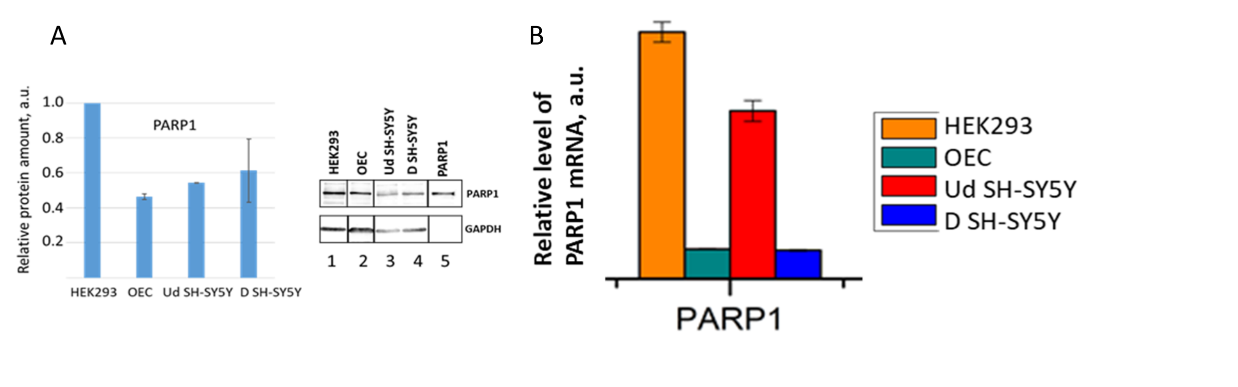


Figure S12. Data of WB analysis (A) and PARP1 mRNA levels (qPCR) (B). Experiments were done as described in the S.1.2.2. and S.1.2.3. sections. The data from three independent experiments are shown as the means ±S.D.

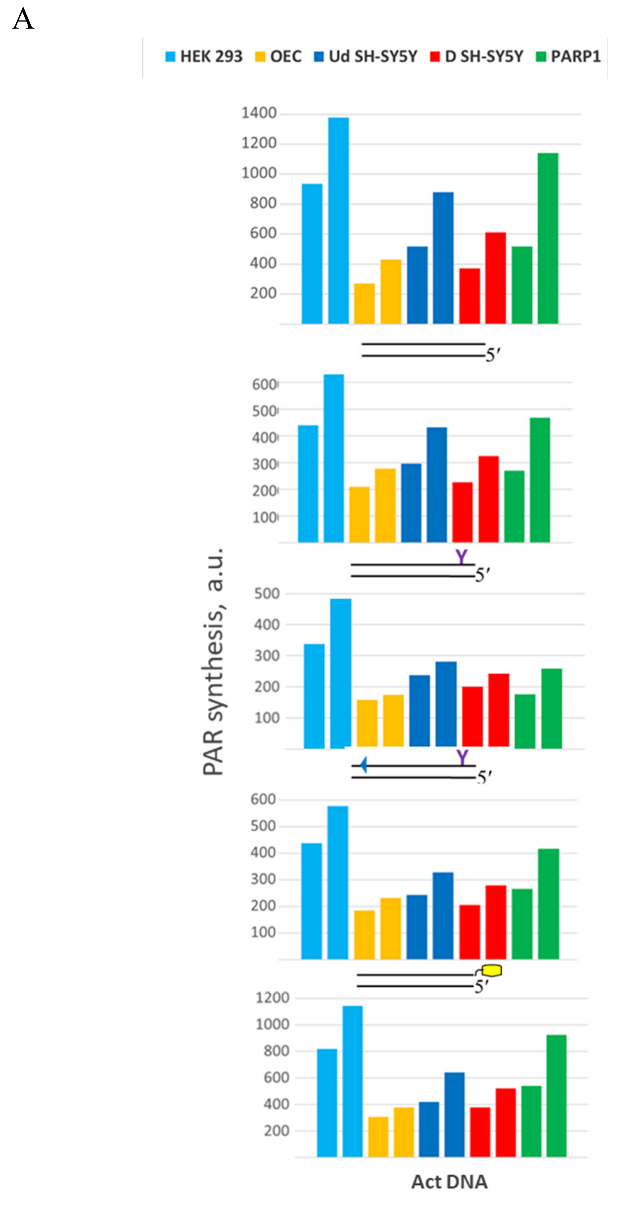


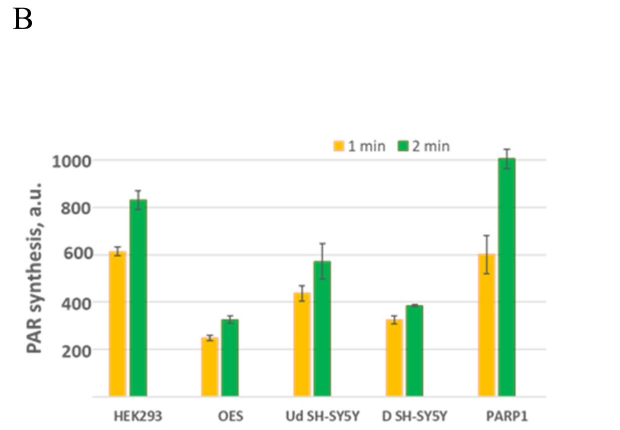


Figure S13. Activating properties of DNAs in the PAR synthesis reaction catalysed by endogenous PARPs of WCEs

The efficiency of PAR synthesis was estimated as described 'Activating properties of LS-DNA in the PAR synthesis'. A. Concentrations of the main reagents were 0.25 mg/ml of WCE proteins, 7.5 nM PARP1, 0.6 $A_{260}$/ml of activated DNA. Reaction times were 1.0 min and 2 min. B. Concentrations of the main reagents were 0.25 mg/ml of WCE proteins, 7.5 nM PARP1, 0.6 $A_{260}$/ml of activated DNA and 0.1 µM other DNAs. Reaction times were 0.5 min and 1 min for panel A and 1 and 2 min for panel B.
